# Secreted Factors by Anaplastic Thyroid Cancer Cells Induce Tumor-Promoting M2-like Macrophage Polarization through a TIM3-Dependent Mechanism

**DOI:** 10.3390/cancers13194821

**Published:** 2021-09-26

**Authors:** Cinthia Carolina Stempin, Romina Celeste Geysels, Sunmi Park, Luz Maria Palacios, Ximena Volpini, Claudia Cristina Motran, Eva Virginia Acosta Rodríguez, Juan Pablo Nicola, Sheue-yann Cheng, Claudia Gabriela Pellizas, Laura Fozzatti

**Affiliations:** 1 Departamento de Bioquímica Clínica, Facultad de Ciencias Químicas, Universidad Nacional de Córdoba, Córdoba 5000, Argentina; cstempin@unc.edu.ar (C.C.S.); romina.geysels@unc.edu.ar (R.C.G.); luzmpalacios@mi.unc.edu.ar (L.M.P.); ximenavolpini@unc.edu.ar (X.V.); cmotran@unc.edu.ar (C.C.M.); eva.acosta@unc.edu.ar (E.V.A.R.); juan.nicola@unc.edu.ar (J.P.N.); claupellizas@unc.edu.ar (C.G.P.); 2 Centro de Investigaciones en Bioquímica Clínica e Inmunología, Conicet, Córdoba 5000, Argentina; 3 Laboratory of Molecular Biology, Center for Cancer Research, National Cancer Institute, National Institutes of Health, Bethesda, MD 20892, USA; SUNMIP@yuhs.ac (S.P.); chengs@mail.nih.gov (S.-y.C.)

**Keywords:** anaplastic thyroid cancer, Tumor-associated macrophages, M2-like macrophage polarization, TIM3

## Abstract

**Simple Summary:**

Among the different types of thyroid cancer, anaplastic thyroid cancer (ATC) is one of the most aggressive tumors. Characterized for its undifferentiated cells, it spreads quickly to distant organs and does not respond well to standardized therapy. Therefore, there is a critical need to identify new targets that can be translated into therapeutic approaches. ATCs are heavily infiltrated by Tumor-Associated Macrophages (TAMs), and its infiltration density is associated with decreased survival. However, the functional role of TAMs in ATC is still unclear. Our results provide valuable insights into the processes in which soluble factors produced by ATC cells induce M2-like polarization of human monocytes through T cell immunoglobulin and mucin-domain containing protein-3 (TIM3). TIM3 in TAMs should now be further evaluated as a possible potential novel target for treating ATC.

**Abstract:**

Anaplastic thyroid cancer (ATC) is a highly aggressive type of thyroid cancer (TC). Currently, no effective target treatments are available that can improve overall survival, with ATC representing a major clinical challenge because of its remarkable lethality. Tumor-associated macrophages (TAMs) are the most evident cells in ATCs, and their high density is correlated with a poor prognosis. However, the mechanisms of how TAMs promote ATC progression remain poorly characterized. Here, we demonstrated that the treatment of human monocytes (THP-1 cells) with ATC cell-derived conditioned media (CM) promoted macrophage polarization, showing high levels of M2 markers. Furthermore, we found that STAT3 was activated, and this was correlated with an increased expression and secretion of the inflammatory cytokine interleukin-6. Remarkably, the M2-like macrophages obtained revealed tumor-promoting activity. A cytokine array analysis demonstrated that M2-like macrophage-derived CM contained high levels of TIM3, which is an important immune regulatory molecule. Consistently, TIM3 expression was up-regulated in THP-1 cells cultured with ATC cell-derived CM. Moreover, TIM3 blockade significantly reversed the polarization of THP-1 cells induced by ATC cell-secreted soluble factors. We validated the clinical significance of the TIM3 in human TC by analyzing public datasets and found that the expression of TIM3 and its ligand galectin 9 was significantly higher in human TC tissue samples than in normal thyroid tissues. Taken together, our findings identified a new mechanism by which TIM3 induces tumor-promoting M2-like macrophage polarization in TC. Furthermore, TIM3 interference might be a potential tool for treatment of patients with ATC.

## 1. Introduction

Thyroid cancer (TC) is one of the most common endocrine tumors [1], with TC having been found to include several subtypes in terms of different outcomes and therapies [2,3]. Of these, papillary thyroid cancer (PTC), one of its most frequent varieties, has a good prognosis with a very good overall survival rate [4]. In contrast, anaplastic thyroid cancer (ATC), although being a very rare type of cancer, is an extremely aggressive disease with the poorest prognosis due to low responsiveness to most therapies [5,6,7]. In fact, ATC patients have a median overall survival of about 6 months [7]. The standard treatment for ATC includes a combination of surgery, chemotherapy, and radiotherapy, with limited efficacy [7]. Recently, a combination therapy of dabrafenib (BRAF inhibitor) with trametinib (MEK inhibitor) showed promising results for treatment of ATC patients [8]. However, drug resistance and tumor recurrence often develop. On the other hand, immunotherapy for ATC patients is currently an active research area. While checkpoint inhibitors are approved for a variety of solid tumors, none have been approved specifically for ATCs [7]. Therefore, a better understanding of the molecular mechanisms is urgently needed in order to be able to identify novel molecular targets that can be used for ATC therapy and thereby improve the outcomes of these patients.

Significant advances have been made to identify the genetic driver mutations implicated in TC progression [4,9]. However, genetic alterations within tumor cells are not solely what determines tumor progression, and it has been shown that the interplay between tumor cells and their surrounding tumor microenvironment (TME) significantly supports tumor growth [10,11]. The TME comprises a variety of stromal cells, including fibroblasts, immune cells, and endothelial cells, among others, with therapeutic strategies targeting the TME gaining increasing attention [11,12,13].

We have recently described the dynamic interaction that exists between tumor cells and human fibroblasts, which, in turn, fosters thyroid cancer progression [14,15]. Macrophages in the TME are referred to as Tumor-Associated Macrophages (TAMs) and are another one of the major components of the TME, promoting crucial steps in tumor progression, including tumor cell proliferation, invasion, metastasis, angiogenesis, and the suppression of adaptive anti-tumor immunity [16,17]. The pro-tumoral behavior of TAMs has made them an attractive therapeutic target. Targeting strategies include the specific depletion of TAMs and functional/phenotypic reprogramming, among others [18]. Interestingly, ATC is heavily infiltrated with TAMs, representing more than 50% of the tumor volume [19,20,21], with the presence of a high density of TAMs in ATC correlating with a decreased cancer-related survival [19,22]. Therefore, TAMs are emerging as promising therapeutic targets for combinatorial therapy in this extremely virulent form of cancer, which is almost invariably fatal. However, it is largely unknown how the crosstalk between cancer cells and TAMs promotes ATC progression.

Macrophages, a heterogeneous group of cells, can exhibit a spectrum of phenotypes including classically activated M1 macrophages at one extreme and alternatively activated M2 macrophages at the other extreme of the spectrum. M1-like macrophages are associated with pro-inflammatory/anti-tumor responses, whereas M2-like macrophages are involved in anti-inflammatory/pro-tumor responses [23]. TAMs originate from circulating monocytes, derived mainly from the bone marrow [24]. Upon reaching tumors, monocyte-derived macrophages receive local cytokine and inflammatory signals from the TME [24]. Interestingly, in advanced solid cancers, the secretion of cytokines and tumor signals typically polarizes monocyte-derived macrophages toward the M2-like phenotype linked with tumor progression and the suppression of tumor-specific immunity.

Considering their plasticity, reprogramming the polarization of TAMs may influence the functions of TAMs, with this concept having attracted great attention [18,24,25]. However, the function and polarization of TAMs in ATC remains poorly understood. Therefore, in the present study, we investigated how factors secreted from ATC cells influence macrophage function and phenotype and also explored the potential mediators of their effects. We showed that conditioned media (CM) collected from ATC cells induced macrophage polarization toward a tumor-promoting M2-like phenotype. It was also demonstrated that T cell immunoglobulin and mucin-domain-containing protein-3 (TIM3) expression in TAMs plays a role in this process. In line with these observations, we found that TIM3 expression levels increased in human TC. Thus, TIM3 in TAMs is a promising target in human ATC immunotherapy.

## 2. Materials and Methods

### 2.1. Cell Culture

The THP1 cell line (human monocytes) and human T lymphocyte (Jurkat) cells were obtained from American Type Culture Collection (ATCC) and confirmed as mycoplasma-free. THP-1 cells and Jurkat cells were grown in RPMI 1640 medium (Gibco, Thermo Fisher Scientific, Waltham, MA, USA). Human ATC cells, 8505c and KTC-2 (passage number ranged from 10 to 15), were authenticated by short-tandem repeat (STR) profiling analysis by the Science Cordoba Agency (CEPROCOR, Córdoba, Argentina), as previously described [14]. ATC cells were cultured in Dulbecco’s modified Eagle’s medium (DMEM) (Gibco, Thermo Fisher Scientific, Waltham, MA, USA)), as previously reported [14]. The PTC-derived TPC-1 and K1 cell lines were obtained from the University of Colorado Cancer Center Cell Bank and maintained in RPMI 1640 medium (Gibco, Thermo Fisher Scientific, Waltham, MA, USA). All cell lines were cultured in medium supplemented with 10% fetal bovine serum (FBS, Hyclone, Logan, UT, USA), penicillin/streptomycin, and L-glutamine (Gibco, Thermo Fisher Scientific, Waltham, MA, USA).

### 2.2. Monocyte–Macrophage Differentiation

To generate monocyte-derived M2 macrophages, THP-1 monocytes were supplemented for 48 h with 5 ng/mL phorbol myristate acetate (PMA, Sigma-Aldrich, St. Louis, MO, USA) in 24-well culture plates with cell suspension (1 × 10^6^ cells). Then, the media were supplemented with 40 ng/mL recombinant human IL-4 (#204-IL; R&D Systems, Minneapolis, MN, USA) and cultured for an additional 18 h, 24 h, 48 h, and 72 h to achieve M2 macrophage polarization.

### 2.3. Conditioned Media (CM) Harvesting

**Figure 1:**
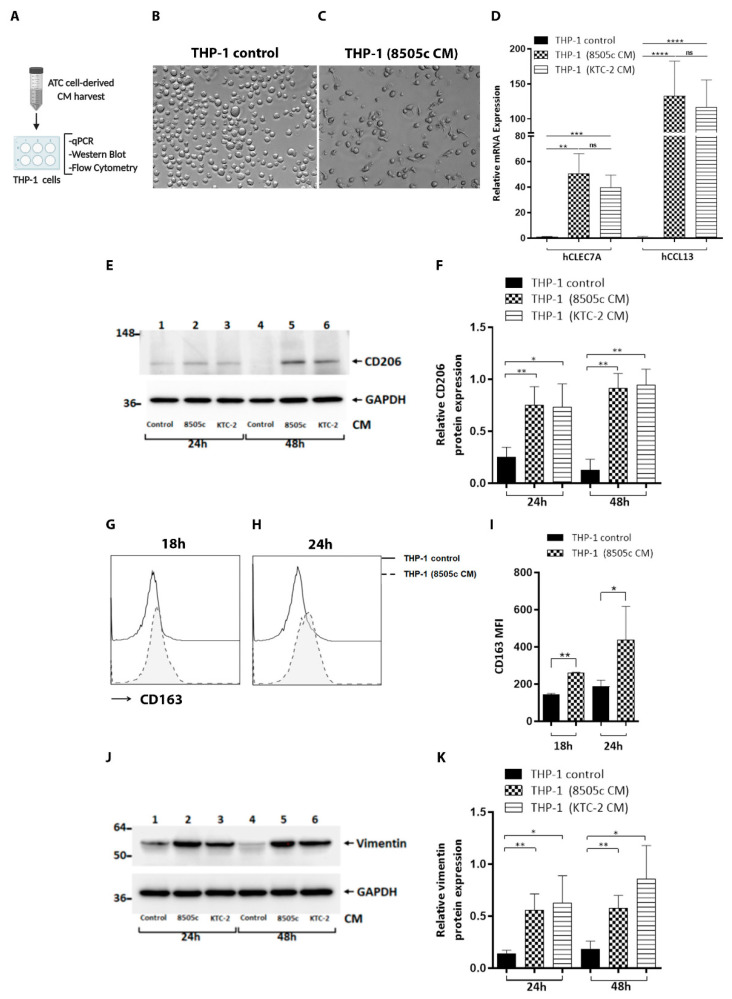
Differentiation of THP-1 monocytes into M2-like macrophages. ATC cell-derived CM induced the expression of M2 macrophage markers in human monocytes. (**A**) Schematic representation of CM preparation from ATC cells and treatment of THP-1 cells with complete media containing 5% FBS or with 100% CM of ATC cell-derived CM for different periods of time (18 h, 24 h, 48 h), and a variety of parameters were analyzed. (**B**,**C**) Soluble factors derived from ATC cells induced morphological changes in THP-1 cells. Monocytes were incubated with complete media containing 5% FBS or treated with ATC cell-derived CM for 24 h. Representative bright-field imaging of THP-1 control (**B**) or treated with 8505c-derived CM, THP-1 (8505c CM) (**C**). (**D**) THP-1 cells treated with ATC cell-derived CM for 24 h showed a significant increase of mRNA expression of hCLEC7A or Dectin-1 and hCCL13 (both markers for M2 macrophages), compared with complete media, measured by RT-qPCR. (**E**,**F**) Immunoblot analysis of CD206 in THP-1 cells grown under normal conditions (THP-1 control) or exposed to derived from ATC cells for 24 h and 48 h (**E**). Quantification of relative expression of CD206 protein in human monocytes after using GAPDH as loading control (**F**). Uncropped Western Blot is shown in Figure S7. (**G**–**I**) Flow cytometry analysis showed significant induction of CD163 in THP-1 cells treated with 8505c-derived CM for 18 h (**G**) and 24 h (**H**) compared to THP-1 control. Representative histograms (**G**,**H**) and quantification (**I**) are shown. (**J**,**K**) Immunoblot analysis of vimentin in THP-1 cells grown under normal conditions (THP-1 control) or exposed for 24 h and 48 h to CM derived from ATC cells (**J**). Quantification of relative expression of vimentin protein in human monocytes after using GAPDH as loading control (**K**). Full Western Blot is shown in Figure S7. Data are expressed as mean ± SD. * *p* < 0.05, ** *p* < 0.005, *** *p* < 0.0005, **** *p* < 0.0001. ns, not significant. Created with BioRender (Online Software, San Francisco, CA, USA).

The CM preparation from ATC cell lines was performed as previously described [14]. Briefly, ATC cells (8505c and KTC-2) were seeded (1 × 10^6^ cells) on 100 mm tissue culture plates in DMEM 10% FBS. Then, 48 h later, the media were replaced with 5% FBS. After an additional 48 h, CM were collected and clarified by centrifugation (2000 rpm for 10 min). From here on, CM from 8505c and KTC-2 cells (ATC cell-derived CM) are referred to as 8505c CM and KTC-2 CM, respectively. The THP-1 cells (1 × 10^6^ cells/well) were cultured in complete media containing 5% FBS (THP-1 control) or treated with 100% CM of ATC cells (THP-1 (8505c CM) and THP-1 (KTC-2 CM) for different periods of time, and a variety of parameters were analyzed (Figure 1A).

To obtain CM derived from activated macrophages by ATC cell-secreted soluble factors, THP-1 cells (1 × 10^6^ cells/mL) were cultured for 48 h with complete media containing 5% FBS or ATC cell-derived CM. The medium was discarded, and the cells were washed with phosphate-buffered saline (PBS) before being incubated with fresh medium containing 5% FBS. After 48 h, the CM were harvested, centrifuged, and stored at −80 °C until use. CM derived from THP-1 cells cultured with complete media containing 5% FBS or ATC cell-derived CM are from here on referred to as CM THP-1 control, CM THP-1 (8505c CM), and CM THP-1 (KTC-2 CM), respectively. TPC-1 and K1 cells were treated with 100% of the CM THP-1 control or CM THP-1 (8505c CM) and CM THP-1 (KTC-2 CM).

### 2.4. RNA Isolation and Quantitative Real-Time RT-PCR

Total RNA purification, cDNA synthesis, and quantitative PCR (qPCR) were performed as previously described [14,26,27]. The primers used are listed in Table S1.

### 2.5. Flow Cytometry

Cells were harvested, washed with PBS, and resuspended in 1% FBS in PBS. Then, cells were incubated with the following fluorochrome-conjugated monoclonal antibodies for 20 min at 4 °C: APC anti-human CD366 (TIM3, #345011), APC anti-human CD206 (#321109), and PE anti-human CD163 (#333605), which were all obtained from BioLegend (San Diego, CA, USA) and used at 1:10 and 1:20 dilutions. Then, cells were analyzed in a FACS Canto II flow cytometer (BD Biosciences, San Jose, CA, USA) using FlowJo software [14].

### 2.6. Western Blot Analysis

Whole-cell lysates were prepared as previously described [14,28]. The protein samples (20–30 µg) were analyzed by Western blot, which was also described previously [14]. Antibodies, which included CD206 (sc-58986), Cyclin D1 (sc-450), and E-Cadherin (sc-7870) from Santa Cruz Biotechnology, were utilized according to the manufacturers’ manuals include and used at a 1:200 dilution. Anti-inducible nitric oxide synthase (iNOS/NOS Type II, #610333) was obtained from BD Biosciences and used at a 1:1000 dilution. Anti-phosphorylated STAT3 (Tyr705, #9131), total STAT3 (#4904), vimentin (#5741), and GAPDH (#2118), were all obtained from Cell Signaling and used at a 1:1000 dilution. Band intensities were quantified by using NIH IMAGE software (ImageJ 1.50i; NIH, Bethesda, MD, USA).

### 2.7. Determination of Interleukin-6 (IL-6) by Enzyme-Linked Immunosorbent Assay (ELISA)

Culture supernatants were harvested, clarified by centrifugation, and frozen for subsequent determination of the IL-6 concentration by ELISA according to the manufacturer’s instructions (#430501, BioLegend, San Diego, CA, USA), as previously described [14].

### 2.8. In Vitro Cell Proliferation Assay

The cell proliferation assay was performed as previously described [14]. Briefly, 2 × 10^6^ THP-1 cells/well were incubated with ATC cell-derived CM or DMEM (control) in six-well plates, with each assay carried out in triplicate. The number of viable cells was counted 24 h and 48 h after treatment using a cell counter (Countless II, ThermoFisher Scientific, Waltham, MA, USA).

### 2.9. Flow Cytometric Analysis of the Cell Cycle

The cell cycle analysis was carried out as previously described [29]. THP-1 cells were treated with ATC cell-derived CM or DMEM (control) for 24 h, after which cells were collected and fixed with cold 70% ethanol and stored at −20 °C for at least 2 h. Then, the cells were washed in PBS buffer and incubated with 1 µg/mL 4′, 6-diamidino-2-phenylindole (DAPI) solution for 10 min before being analyzed. The cell cycle profiles were determined using flow cytometry (LSR Fortessa II, BD Bioscience) and analyzed with FlowJo.

### 2.10. Wound-Healing Assay

For the monolayer scratch-induced migration assays, TPC-1 or K1 cells were seeded in 24-well plates at a density of 1 × 10^5^ cells/well and grown until they reached a confluence of ≈70%. A scratch was made using a sterile 20 μL pipette tip for each well of the cells treated with 100% CM THP-1 control and CM THP-1 8505c CM or CM THP-1 KTC-2 CM. Images of cells with a scratch were captured at the time of the scratch (0 h) and 24 h after scratching, which were quantified using ImageJ (Fiji ImageJ 2.0, NIH, Bethesda, MD, USA).

### 2.11. CFSE-Based Proliferation Assay

Jurkat cells were labeled with carboxyfluorescein diacetate succinimidyl ester (CFSE) as previously described [26]. Labeled cells were mixed for 18 h with THP-1 cells that had been previously treated for 48 h with ATC cell-derived CM at a ratio of 0.5:1. Data were acquired on a LSR Fortessa X-20 flow cytometer (BD Biosciences, San Jose, CA, USA) and analyzed using FlowJo software.

### 2.12. Zymography

Gelatin zymography was performed to determine the gelatinolytic activity in the CM (40 μL). Gels containing 1.5% gelatin were run for ≈2 h. Then, after electrophoresis, the gels were incubated in 2.5% Triton X-100 for 40 min to remove the SDS. Then, these were equilibrated in developing buffer (50 mM Tris-HCl, 200 mM NaCl, 9 mM CaCl_2_, 0.02% Brij 35) and incubated overnight at 37 °C. The gels were stained in 45% methanol and 5% acetic acid with 0.125% Coomassie R250 and destained in 10% acetic acid and 25% ethanol. The gelatinolytic activities were visualized as a clear band against a dark background.

### 2.13. TIM3 Blockade

The TIM3 pathway was interfered with by TIM3 blocking antibody, with the THP-1 cells being incubated with 10 μg/mL of TIM3 blocking antibody (#345004, BioLegend, San Diego, CA, USA) or IgG isotype control antibody (#400153, BioLegend, San Diego, CA, USA) for 24 h. Then, the CLEC7A, CCL13, and IL-6 mRNA levels were measured by RT-qPCR or ELISA.

### 2.14. Human XL Cytokine Array

CM from the THP-1 cells were collected 48 h after incubation with ATC cell-derived CM. The supernatant was analyzed by the Proteome Profiler Human XL Cytokine Array Kit (#ARY022B, R&D Systems, Minneapolis, MN, USA), according to the manufacturer’s instructions. Densitometry analysis was carried out using Image J (ImageJ 1.50i; NIH, Bethesda, MD, USA) to quantify the pixel density of each spot.

### 2.15. Nitrite Assay

Culture supernatants were harvested, clarified by centrifugation, and frozen for subsequent determination of nitrite as previously described [26,30].

### 2.16. Gene Expression Analysis

Microarray datasets, generated on the Affymetrix GPL570 platform containing ATCs (GSE29265, GSE33630, GSE76039 and GSE65144) [9,31,32] and normal thyroid tissue (GSE3467, GSE3678, GSE6004, GSE29265, GSE33630, GSE53157, GSE35570, GSE60542) [33,34,35,36,37], were downloaded from the NCBI Gene Expression Omnibus database (http://www.nibi.nih.gov/geo/ (accessed on 16 March 2020)) and processed in R version 3.6.3 (https://www.r-project.org/ (accessed on 15 March 2020)). Data were normalized using the GeneChip Robust Multiarray Averaging method, and a matrix of 171 normal tissues, and 52 ATCs was generated. Genes were annotated using Affymetrix Human Genome U133 Plus 2.0 Array annotation data (Affymetrix, Santa Clara, CA, USA).

### 2.17. Statistical Analysis

Analysis of intergroup differences was performed using one-way analysis of variance (ANOVA), which was followed by Turkey’s test. For the analysis of differences between two groups, a statistical analysis was carried out using the Student’s *t*-test or Mann–Whitney test with GraphPad Prism 6.01 (GraphPad Software, San Diego, CA, USA). Values of *p* < 0.05 were considered to be statistically significant.

## 3. Results

### 3.1. Phenotypic Reprogramming of Human Monocytes Induced by Soluble Factors Secreted by ATC Cells

ATCs are characterized by having a high density of M2-like TAMs [9,19,20,21]. However, the mechanisms that participate in the control of the phenotypic and the functional alterations of TAMs in ATC remain poorly characterized. In advanced solid cancers, the secretion of cytokines and tumor signals are commonly thought to recruit and polarize monocytes toward the M2-like macrophage phenotype linked with tumor progression and the suppression of tumor-specific immunity [24,38,39]. To test this hypothesis using an in vitro model, we explored whether the soluble factors secreted by ATC cells can modulate monocyte-derived macrophage phenotypes. To this end, an acute monocytic leukemia cell line, THP-1, was used as a human model of monocytes. This cell line has been extensively used to study monocyte/macrophage functions, mechanisms, and signaling pathways, and it has become a common model for estimating the modulation of monocyte and macrophage activities [40]. We first set up a model of M2 macrophages. As it has been previously shown that the exposure of the THP-1 cell line to phorbol myristate acetate (PMA) and hIL-4 drives M2 polarization, this was assessed by measuring the expression of several classical M2 markers (CCL13, C-type lectin-like receptor (CLEC7A or Dectin-1) and CD206 or Mannose receptor C type (MRC1)) as suggested in several studies [23,38,41,42]. We found that the mRNA expression of CCL13, CLEC7A, and CD206 (Figure S1A–C) were higher in M2 macrophages polarized by hIL-4 and PMA, compared with controls and PMA alone, at all the times analyzed. Macrophage M2 polarization was also evaluated at the protein level of CD206 (Figure S1D,E) and CD163 (Figure S1F,G) by FACS analysis, with the expression of CD206 being observed to be clearly increased in the THP-1 cells treated with hIL-4 and PMA, compared with controls and PMA alone (Figure S1D, with Figure S1E showing the quantitative data). Similar results were also obtained for CD163 (Figure S1F,G (quantitative analysis)). Taken together, these observations confirmed the M2 macrophage phenotype.

To investigate whether ATC cells produce soluble factors that influence monocyte activation and functional polarization, THP-1 cells were exposed to conditioned media (CM) produced by ATC cells (8505c and KTC-2 cells), and the phenotypes of the macrophages were analyzed. We prepared CM from ATC cells, as described previously [14] (Figure 1A), and it was found that ATC cell-derived CM strongly influenced the phenotype of the THP-1 cells. Human monocyte-derived macrophages increased their adherence and changed their morphology after treatment with 8505c cell-derived CM during 24 h (Figure 1C) compared to the monocyte controls (Figure 1B). In addition, THP-1 cells exposed to ATC cell-derived CM displayed a M2-like macrophage phenotype, expressing higher levels of CLEC7A and CCL13 mRNA than the THP-1 controls (Figure 1D). Western blot revealed a significant increased abundance of CD206 in THP-1 cells treated for 24 h and 48 h with CM derived from ATC cells, compared with THP-1 cell controls ((Figure 1E, compare lanes 1 (24 h) and 4 (48 h) (THP-1 control) with lanes 2 (24 h) and 5 (48 h) (8505c cell-derived CM) and 3 (24 h) and 6 (48 h) (KTC-2 cell-derived CM)), with Figure 1F showing a quantitative comparison of the band intensities from Figure 1E. Flow cytometry revealed that the M2 macrophage marker CD163 was also increased in monocytes incubated for 18 h (Figure 1G) or 24 h (Figure 1H) with CM derived from 8505c cells compared with complete media, and Figure 1I shows the quantitative data. Vimentin is the most abundant intermediate filament protein, which supports cellular structures in different types of cells [43]. Some studies have reported that activated macrophages increase the expression of vimentin [44]. Therefore, we investigated whether ATC cell-derived CM could alter the expression levels of this protein. Consistent with the activated phenotype, Western blot analysis revealed an up-regulation of vimentin in THP-1 cells after treatment for 24 h and 48 h with ATC cell-derived CM (Figure 1J, compare lanes 1 (24 h) and 4 (48 h) (controls) with lanes 2 and 3 (24 h) and 5 and 6 (48 h)), with the quantitative data of the band intensities being shown in Figure 1K. Next, we examined the levels of M1 macrophage markers. It is known that M1 polarized macrophages are characterized by high expression of inducible nitric oxide synthase (iNOS) [45]. Therefore, we assessed whether ATC cell-derived CM could alter the expression levels of this protein. As shown in Figure S2, the expression of iNOS was not up-regulated in THP-1 cells after treatment for 24 h with 8505c cell-derived CM or KTC-2 cell-derived CM (Figure S2A, compare lanes 1 (THP-1 control) with lanes 2 (8505c cell-derived CM) and 3 (KTC-2 cell-derived CM)) compared to the monocyte controls. After induction, iNOS produces high amounts of nitric oxide (NO) [45]. Therefore, we examine NO production by Griess assay. Consistently, we failed to detect NO generation in THP-1 cells treated with CM of ATC cells for 24 h (Figure S2B), supporting the notion that iNOS was not induced in THP-1 cells by the effect of ATC cell-derived CM. Taken together, these data suggest that tumor cells secrete soluble factors that influence the activation and polarization of monocytes and are able to switch these, most probably into M2-like macrophage phenotypes.

### 3.2. ATC Cell-Derived CM Significantly Decreased Proliferation of Human Monocytes and Delayed Their Cell Cycle at the G0/G1 Stage

**Figure 2:**
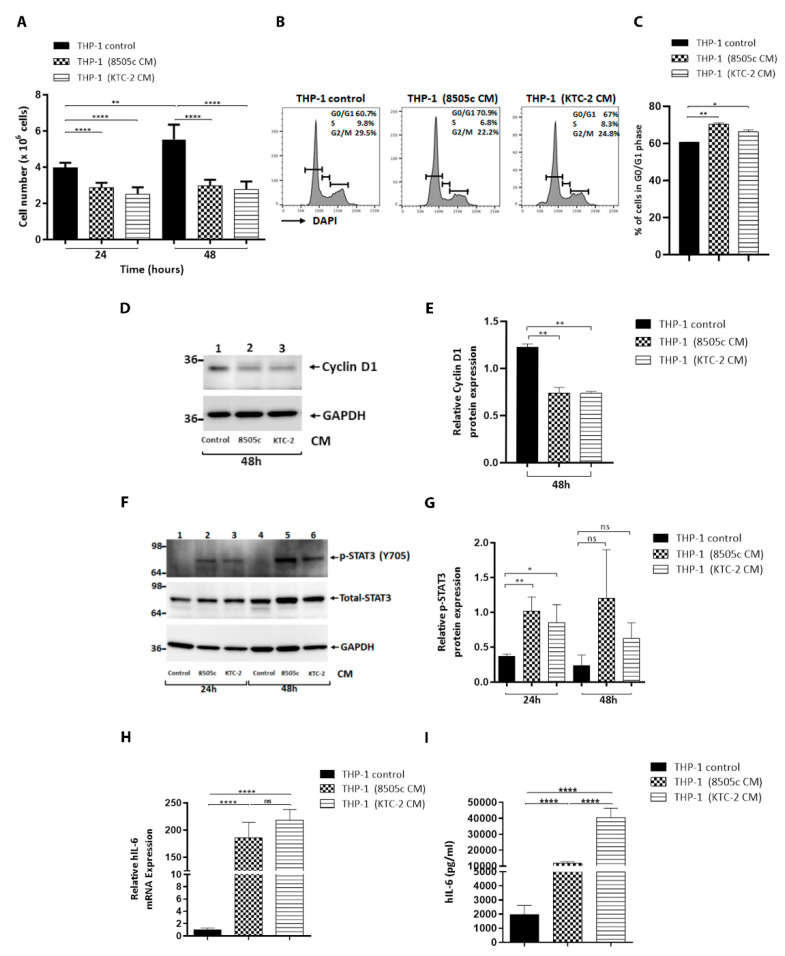
ATC cell-derived CM decreased proliferation of human monocytes. (**A**) Proliferation of THP-1 cells incubated with complete media containing 5% FBS (THP-1 control) or 100% 8505c-derived CM and 100% KTC-2-derived CM for 24 h or 48 h, estimated by cell counting. (**B**) Cell cycle profiles of THP-1 cells incubated with complete media containing 5% FBS (THP-1 control) or treated for 24 h with ATC cell-derived CM by flow cytometry analysis. The average percentages of cell populations of different cell cycle phases are shown in the upper-right corner. (**C**) The graphs showed the percentage of the G0/G1 phase for control and treatment with ATC cell-derived CM in THP-1 cells. The cell cycle phase was quantified by DAPI staining, followed by FACS analysis in THP-1 cells. (**D**,**E**) Immunoblot analysis of cyclin D1 in THP-1 cells grown under normal conditions (THP-1 control) or exposed for 48 h to CM derived from ATC cells (**D**). Quantification of relative expression of cyclin D1 protein in human monocytes after using GAPDH as loading control (**E**). Full Western Blot is shown in Figure S7. Human monocytes activated by ATC cell-derived CM promoted IL-6 levels via the induction of STAT3 phosphorylation (**F**,**I**). (**F**) Immunoblot for p-STAT3 from THP-1 cells incubated with complete media containing 5% FBS (THP-1 control) or treated with CM derived from ATC cells, 8505c and KTC-2, for 24 h and 48 h. The figure shows a representative Western blot of four independent experiments. The lower panel was the corresponding loading control using GAPDH. (**G**) Quantification of relative expression of p-STAT3/Total STAT3 proteins in human monocytes. The same blot was stripped and re-blotted using anti-Total STAT3 antibody. Uncropped Western Blot is shown in Figure S7. (**H**) Expression levels of IL-6. mRNA levels by RT-qPCR in control and THP-1 cells incubated with complete media containing 5% FBS (THP-1 control) or treated for 24 h with ATC cell-derived CM. (**I**) Secreted IL-6 was measured by ELISA. THP-1 cells were incubated with complete media containing 5% FBS (THP-1 control) or treated for 48 h with ATC cell-derived CM. The media were replaced by fresh media. After 48 h, the supernatant was harvested and used for ELISA assays. Data are expressed as mean ± SD. * *p* < 0.05, ** *p* < 0.005, **** *p* < 0.0001. ns, not significant.

Previous studies have shown that the in situ proliferation of monocytes and macrophages can contribute to the accumulation of TAMs. We examined the effect of the ATC cell-derived soluble factors on the proliferation of human monocytes by counting the cells. As shown in Figure 2A, the THP-1 cell number was significantly decreased by treatment with ATC cell-derived CM at 24 h and 48 h. This reduction in the cell number suggests that the cell cycle progression of the THP-1 cells could be delayed. Thus, we carried cell cycle analysis by flow cytometry, Figure 2B displaying the profiles of the G0/G1, S, and G2/M cell cycles for THP-1 cells after treatment with ATC cell-derived CM. The quantitative data indicate that the entry of human monocytes from the G0/G1 phase and S phase was significantly delayed (Figure 2C). These findings prompted us to evaluate the protein abundance of the key cell cycle regulator cyclin D1. In agreement, the Western blot assay showed that the protein level of Cyclin D1 was lower in THP-1 cells incubated for 48 h with ATC cell-derived CM, compared with complete media (Figure 2D, compare lanes 1 (controls) with lanes 2 and 3), with the quantitative data of the band intensities being shown in Figure 2E. Taken together, these results indicate that soluble factors produced by ATC cells significantly decreased the proliferation of THP-1 cells by delaying the cell cycle progression, which was probably due to switching from proliferation to differentiation.

### 3.3. THP-1 Cells Activated by ATC Cell-Derived CM Promoted IL-6 Levels via the Induction of STAT3 Phosphorylation

The signal transducer and activator of the transcription 3 (STAT3) pathway has been extensively described as being an important signaling axis in macrophage biology [46,47]. It has long been known that the activation of STAT3 is essential for macrophage differentiation toward the M2 phenotype [48]. Therefore, we next investigated the effects of ATC cell-derived CM on STAT3 activation in human monocytes. As shown in Figure 2F, STAT3 phosphorylation (p-STAT3) was significantly increased in THP-1 cells by stimulation for 24 h with soluble factors secreted by ATC cells (Figure 2F, compare lanes 1 (control) with lanes 2 (8505c cell-derived CM) and 3 (KTC-2 cell-derived CM)), with Figure 2G showing the quantitative comparison of the band intensities from Figure 2F. Human monocytes were cultured in the presence or in the absence of ATC cell-derived CM for 3, 6, and 24 h. An increase in Tyr 705 phosphorylation of STAT3 was detected at 3 h following the exposure of THP-1 cells to CM derived from ATC cells, compared with complete media (Figure S3A, compare lanes 1 (control) with lanes 2 (8505c-cell derived CM) and 3 (KTC-2 cell-derived CM)).

It has been previously reported that IL-6 binds to its receptor and activates the STAT3 signaling pathway, leading to the transcription of STAT3 target genes [46]. Therefore, we analyzed any changes produced in the levels of this inflammatory marker, which is a known activator of STAT3 signaling, in THP-1 cells. In fact, IL-6 is produced by multiple cell types located within the TME, and our laboratory has previously reported an increase in basal IL-6 levels in 8505c and KTC-2 ATC cells [14]. Interestingly, the expression of IL-6 mRNA was significantly increased in THP-1 cells incubated for 24 h with CM derived from ATC cells compared with complete media (Figure 2H). We further determined the secreted IL-6 in the media by ELISA, and in agreement with the expression changes in the mRNA levels of IL-6, we observed that the treatment of human monocytes for 48 h with ATC cell-derived CM induced a strong release in the secretion of IL-6 at 48 h (Figure 2I). Collectively, our results suggest that the soluble factors released by human ATC cells, including IL-6, promote macrophage activation by stimulating STAT3 signaling. In turn, these activated macrophages somehow further increased the secretion of IL-6 in a feed-forward autocrine loop.

### 3.4. Macrophages Reprogrammed by Soluble Factors Secreted by ATC Cells Exert Tumor-Promoting Functions by Decreasing T Cell Proliferation and Increasing Thyroid Cancer Cell Migration

**Figure 3:**
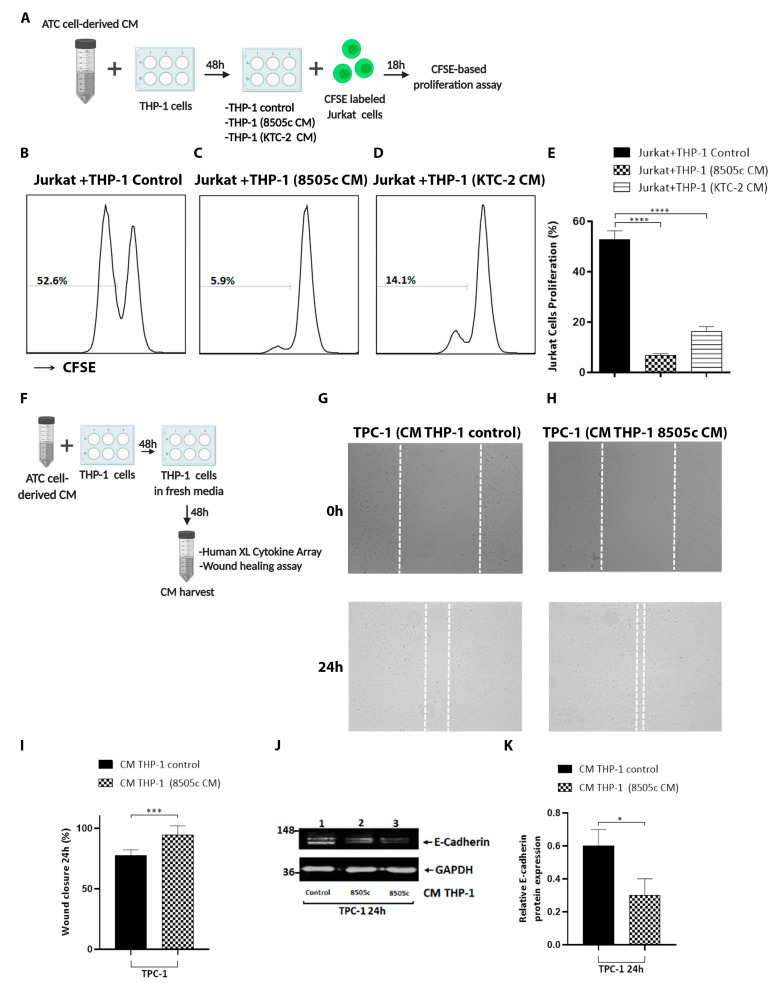
Tumor-educated macrophages by soluble factors secreted by ATC cells exert tumor-promoting functions by decreasing T cell proliferation and increasing thyroid cancer cell migration. (**A**) Schematic of experiment on Jurkat cell proliferation. THP-1 cells were incubated with complete media containing 5% FBS (THP-1 control) or treated with 100% of ATC cell-derived CM for 48 h. T cells were labeled with CFSE and co-cultured with activated macrophages for 18 h. (**B**–**E**) Determination of T cell proliferation by flow cytometry. CFSE-labeled Jurkat cells were co-cultured with THP-1 cells grown under normal conditions (THP-1 control) (**B**) or pretreated for 48 h with 8505c-derived CM (**C**) and KTC-2-derived CM (**D**) at a ratio of 0.5:1 for 18 h. Representative histograms (**B**–**D**) and quantification of percentage of T cells after the co-culture (**E**) are shown. (**F**) Schematic representation of CM preparation from THP-1 cells. Complete media containing 5% FBS 100% or CM from 8505c cells were used for the treatment of THP-1 cells for 48 h. The medium was discarded and then incubated with fresh medium. After 48 h, CM from THP-1 cells were collected and used (100%) to treat TPC-1 cells. (**G**,**H**) Representative cell pictures of wound-healing assay in TPC-1 cells incubated with CM derived from THP-1 control (CM THP-1 (control)) (**G**) or CM derived from activated THP-1 (CM THP-1 (8505c CM)) (**H**) for 24 h. (**I**) Quantitative analysis of wound-induced migration assay from G–H. (**J**) Immunoblot analysis of E-Cadherin in TPC-1 cells after treatment with CM derived from activated THP-1 cells for 24 h. (**K**) Quantification of relative expression of E-cadherin protein in TPC-1 cells after using GAPDH as loading control. Uncropped Western Blot is shown in Figure S7. Data are expressed as mean ± SD. * *p* < 0.05, *** *p* < 0.0005, **** *p* < 0.0001. Created with BioRender.

It has long been known that TAMs play multi-functional roles in tumor progression, including cancer initiation and promotion, metastasis, angiogenesis, and inhibition of the anti-tumor immune responses mediated by T cells [17,47,49]. First, we explored the functional significance of the activation of human monocytes with ATC cell-derived CM on the lymphocyte response. Jurkat T cells were used as a source of T cells, which are an immortalized T-lymphocyte cell line that is often used to study T cell signaling. We performed in vitro T cell interaction experiments and co-cultured CFSE-labeled T cells with THP-1 cells previously treated for 48 h with ATC cell-derived CM for 18 h, after which the T cell proliferation was analyzed by flow cytometry (Figure 3A). It was observed that in vitro T cell proliferation was significantly inhibited during co-culture with THP-1 cells incubated with CM derived from 8505c cells (Figure 3C) and KTC-2 cells (Figure 3D) compared to co-culture with THP-1 cells incubated with complete media (Figure 3B), with Figure 3E showing the quantitative analysis of the proliferating T cells.

Next, we investigated the functional consequences of activation of human monocytes with ATC cell-derived CM on thyroid tumor cell migration. In order to evaluate whether CM from human monocytes activated with ATC cell-derived CM affected thyroid cancer cell motility, wound-healing assays were carried out. To prepare M2-like macrophage-derived CM, THP-1 cells were cultured with CM from 8505c and KTC-2 cells. The media were replaced with fresh media, and after 48 h, the supernatant was collected, centrifuged, and used to treat the TPC-1 and K1 PTC cancer cells (Figure 3F). Cell migration was significantly increased in TPC-1 cells cultured with M2-like macrophage-derived CM (Figure 3G) compared to control THP-1 cell-derived CM (Figure 3H), with Figure 3I displaying the quantitative analysis of the migration assay. Similar results were obtained for K1 cells (Figure S4). Additionally, we examined key regulators of cell motility and migration. Cadherins are responsible for cell–cell adhesion, and epithelial mesenchymal transition (EMT) is a cellular mechanism in which epithelial cadherin (E-Cadherin) is lost during tumor progression. Indeed, E-cadherin was decreased after treatment with M2-like macrophage-derived CM, compared to control TPC-1 cells (Figure 3J, see the quantification in Figure 3K). Taken together, our data indicate that THP-1 cells activated with ATC cell-derived CM demonstrated tumor-promoting activity.

### 3.5. Cytokine Array Analysis Revealed High Levels of TIM3 Expression on M2-like Macrophage-Derived CM

**Figure 4:**
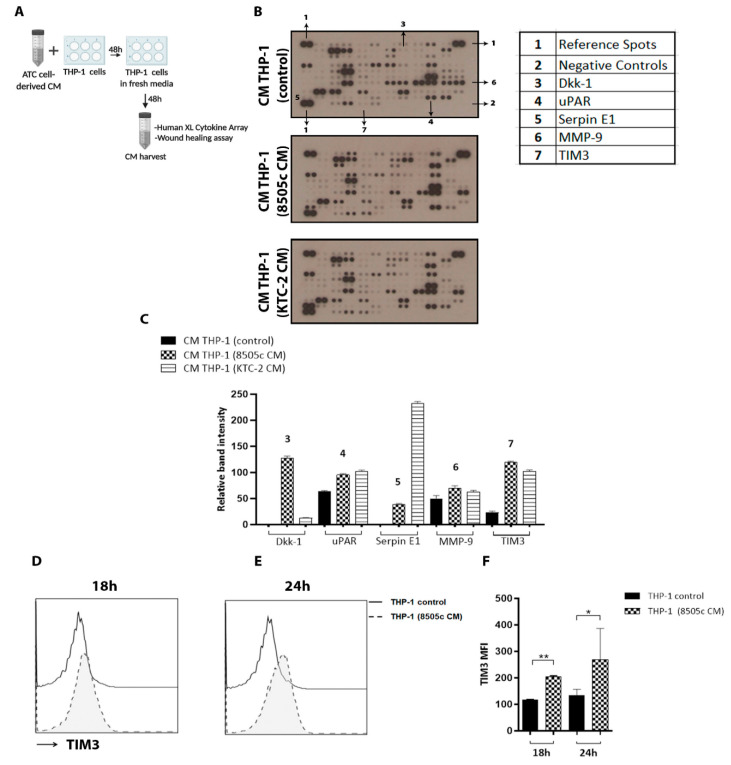
Cytokine array analysis revealed high TIM3 expression on M2-like macrophage-derived CM. (**A**) Schematic representation of CM preparation from THP-1 cells. Complete media containing 5% FBS or 100% CM from 8505c and KTC-2 cells were used for the treatment of THP-1 cells for 48 h. The medium was discarded and then incubated with fresh medium. After 48 h, 100% CM from THP-1 cells were collected and analyzed by the Proteome Profiler Human XL Cytokine Array Kit. (**B**) Cytokine array profiling of CM derived from THP-1 cells. Antibodies for detection of each cytokine were spotted in duplicate. Reference spots and negative controls had six and two spots each, respectively. (**C**) Densitometric analysis revealed that TIM3 was consistently high in both CM THP-1 (8505c CM) and CM THP-1 (KTC-2). (**D**) TIM3 expression was verified by flow cytometry analysis. THP-1 cells treated with 8505c-derived CM for 18 h (**D**) and 24 h (**E**) compared to THP-1 control (incubated with complete media containing 5% FBS). Representative histograms (**D**,**E**) and quantification (**F**) are shown. Data are expressed as mean ± SD. * *p* < 0.05, ** *p* < 0.005. Created with BioRender.

We explored the mechanism by which in vitro-differentiated M2 macrophages exert their pro-tumoral effects and investigated potential soluble mediators of the macrophage effects on tumor promotion and the secretome of THP-1 cells with or without ATC cell-derived CM treatment. To obtain M2-like macrophage-derived CM, we proceeded as depicted in Figure 3F, and the resulting supernatant was analyzed by using the Proteome Profiler Human XL Cytokine Array Kit (Figure 4A). In all, a total of 105 soluble human proteins were screened (Figure 4B). The densitometry analysis results revealed that among others, the levels of Dickkopf-1 (Dkk-1), urokinase plasminogen activator-receptor (uPAR), Serpin E1, matrix metalloproteinase 9 (MMP-9), T cell immunoglobulin, and mucin-domain containing protein-3 (TIM3) were altered in the CM collected from THP-1 cells treated with ATC-cell derived CM (Figure 4C). It is well established that TAMs induce the production of proangiogenic molecules, including matrix metalloproteinases (MMPs). Using gelatin zymography, we validated the levels of MMP-9 activity in the CM collected from THP-1 cells with or without ATC cell-derived CM treatment. As shown in Figure S5, human monocytes treated for 48 h with CM derived from 8505c cells or KTC-2 cells induced an increase in the expression levels of MMP-9 (Figure S5, compare lanes 1 and 2 (controls) with lanes 3 and 4 (CM derived from 8505c cells) and 5 and 6 (CM derived from KTC-2 cells)). However, we did not find any difference in MMP-2 levels (Figure S5).

Of note, the TIM3 level was consistently high in both the CM derived from THP-1 cells treated with ATC cell-derived CM (Figure 4C), with secreted TIM3 being increased by ≈5-fold in the CM from THP-1 cells after treatment with 8505c cell-derived CM and by ≈4.5-fold in the CM from THP-1 cells treated with KTC-2 cell-derived CM (Figure 4C).

The negative immune regulator TIM3 was first identified on activated T effector cells. Previously, reports had indicated that this molecule acts as a negative regulator of T cell activation, and more recent evidence has shown that TIM3 is also expressed on innate immune cells, including monocytes and macrophages [50]. However, we know very little about its role in TAMs in ATC. To confirm the cytokine array findings, TIM3 expression levels were further evaluated by FACS analysis (Figure 4D–F), which confirmed their increase in human monocytes incubated for 18 h or 24 h with CM derived from 8505c cells, compared with complete media (Figure 4D–F (quantitative analyses)). Taken together, these results support the hypothesis that ATC cell-derived soluble factors not only induce human monocyte polarization toward an M2-like phenotype but also up-regulate TIM3 expression.

### 3.6. Blockade of TIM3 Alters Macrophage Polarization

**Figure 5:**
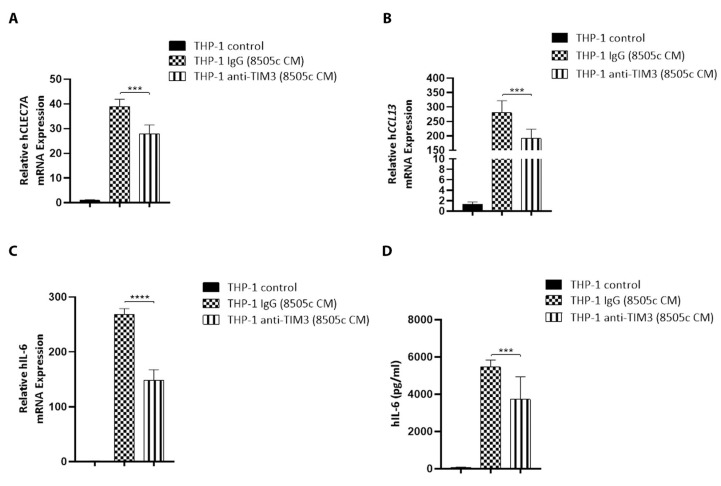
TIM3 modulates M2-like macrophage polarization in vitro. (**A**–**D**) THP-1 cells were incubated with complete media (THP-1 control) or treated with 8505c cell-derived CM (THP-1 (8505c CM)) in the presence of isotype control IgG or anti-TIM3 blocking antibody for 24 h. hCLEC7A (**A**), hCCL13 (**B**), or IL-6 (**C**) mRNA levels were measured by RT-qPCR. (**D**) The supernatant was collected, and IL-6 was detected by ELISA. Data are expressed as mean ± SD. *** *p* < 0.0005, **** *p* < 0.0001.

In some tumor types, increased expression of TIM3 has been associated with disease progression and shorter survival [51]. Interestingly, a recent study demonstrated that TIM3 expression in TAMs is positively correlated with poor survival in patients with hepatocellular carcinoma [52]. Furthermore, previous reports have shown that TIM3 participates in M2-like macrophage polarization [52,53,54]. As we found a strong relationship between the expression of TIM3 and the development of the M2-like macrophage phenotype in human monocytes after treatment with ATC cell-derived soluble factors, we hypothesized that TIM3 could be involved in this process. Thus, we investigated whether TIM3 participates in THP-1 cells polarization by using anti-TIM3 blocking antibodies (anti-TIM3). First, the effects of 8505c cell-derived soluble factors on the expression levels of M2 markers in THP-1 cells were investigated. Consistent with our previous observations, the real-time qPCR results confirmed the up-regulation of CLEC7A, CCL13, and IL6 expression in THP-1 cells incubated for 24 h with 8505c cell-derived CM and control IgG (Figure 5). Interestingly, a combination treatment of 8505c cell-derived soluble factors and anti-TIM3 for 24 h significantly reduced the mRNA expression levels of CLEC7A, CCL13, and IL6 in THP-1 cells (Figure 5A–C). In line with these findings, the secretion of IL-6 was also significantly reduced after TIM3 blockade (Figure 5D). These in vitro data demonstrate that TIM3 is involved in the polarization of human monocytes toward the M2-like phenotype.

### 3.7. M2 Macrophage Markers, TIM3, and Its Ligands Are Up-Regulated in Thyroid Cancer Tissue

**Figure 6:**
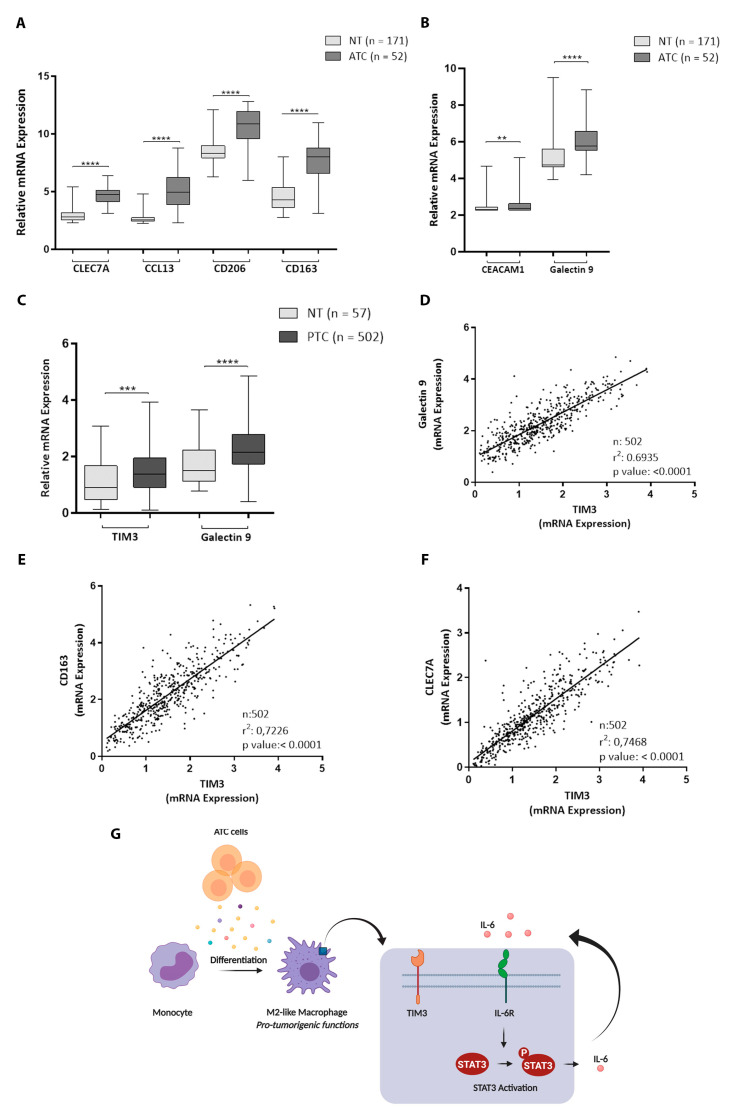
M2 markers, ligands of TIM3 and TIM3 expression in human thyroid tissues. (**A**) Boxplots showing CLEC7A, CCL13, CD206, and CD163 gene expression in normal thyroids (NT) and ATC samples derived from GEO datasets (GSE3467, GSE3678, GSE6004, GSE29265, GSE33630, GSE53157, GSE35570, GSE60542, GSE29265, GSE33630, GSE76039, and GSE65144). Statistical significance by Mann–Whitney test; **** *p* < 0.0001. (**B**) Boxplots showing TIM3 ligands, CEACAM1 and Galectin 9 gene expression in normal thyroids (NT) and ATC samples derived from GEO datasets (GSE3467, GSE3678, GSE6004, GSE29265, GSE33630, GSE53157, GSE35570, GSE60542, GSE29265, GSE33630, GSE76039, and GSE65144). Statistical significance by Mann–Whitney test; ** *p* < 0.005, **** *p* < 0.0001. (**C**–**F**) Analysis of TCGA–THCA data. (**C**) Comparison of the expression of TIM3 and its ligand, Galectin 9, between PTC and NT tissue. Statistical significance by Mann–Whitney test; *** *p* < 0.001, **** *p* < 0.0001. (**D**) Correlation between Galectin 9 and TIM3. (**E**,**F**) Correlation between M2 markers (CD163 and CLEC7A) and TIM3. Secreted factors by ATC cells promote tumor-promoting M2-like macrophage polarization through, at least in part, increasing TIM3 expression (**G**). Soluble factors secreted by ATC cells promote the expression of TIM3 in human monocytes. The enhanced TIM3 expression leads to the polarization of macrophages to the M2 phenotype, resulting in the increased expression of cytokine IL-6 and finally contributing to thyroid cancer progression. Created with BioRender.

ATC is heavily infiltrated with TAMs, which represent more than 50% of the total tumor mass [19,20,21], with TAMs being characterized by presenting an M2-like phenotype. In order to study the TAM phenotypes in ATCs and using the GEO databases, we compared the expression levels of M2 macrophage markers between 171 normal thyroid tissues and 52 ATCs. As shown in Figure 6A, and in line with our in vitro observations, ATCs exhibited a significantly higher expression of CLEC7A, CCL13, CD206, and CD163 compared with normal thyroids. Furthermore, several ligands for TIM3 have been identified, including galectin-9, and most recently carcinoembryonic antigen-related cell adhesion molecule 1 (CEACAM1), which have been implicated in mediating the TIM3 function [50]. Galectin 9 is expressed in multiple cell types, with CEACAM1 also being highly expressed by some tumor cells [50]. In the same cohort, we found a significant increase in the expression levels of both ligands compared with normal thyroid tissue (Figure 6B). Unfortunately, the TIM3 levels could not be evaluated in ATC due to TIM3 not being found in the datasets used. Therefore, the gene expression levels of TIM3 and galectin 9 were analyzed in papillary thyroid carcinomas (PTCs, *n* = 502) and normal tissue (*n* = 57) from The Cancer Genome Atlas (TCGA) datasets. As shown in Figure 6C, the expression levels of TIM3, as well as those of galectin 9 in the PTCs, were significantly higher compared with normal thyroid tissues. In the same cohort, we found a strong correlation between galectin 9 and TIM3 (Figure 6D). In addition, M2 macrophage markers including CD163 (Figure 6E), CLEC7A (Figure 6F), CCL13 (Figure S6A), and CD206 (Figure S6B) were highly correlated with TIM3. Taken together, our results show that M2 macrophage markers and TIM3/galectin-9/CEACAM1 expression levels are increased in thyroid cancer tissues, thereby supporting our in vitro findings.

## 4. Discussion

Increasing attention has been paid to the thyroid TME in recent years. In a previous investigation, we described how human fibroblasts and ATC cells interact to drive TC aggressiveness [14,15]. TAMs are the most abundant cellular component of the TME in ATC, with higher densities of TAMs having been associated with poor survival [19,20,21,22], suggesting that TAMs are likely to promote tumor progression. However, the functional role of TAMs in ATC is still unclear. In the present study, we characterized the interplay between human monocytes and ATC cells and evaluated how this interaction affects TC progression. Furthermore, some mediators involved in these processes were also investigated. This provided evidence suggesting that CM from ATC cells were able to differentiate human monocytes toward the tumor-promoting M2-like phenotype. We observed that following the activation of THP-1 cells with ATC cell-derived CM, there was an increased expression of M2 markers (CLEC7A, CCL13, CD206, and CD163) in addition to the cytokine IL-6, which is in part mediated by activating the STAT3 signaling pathway. In turn, the activated macrophages can foster thyroid cancer progression and build a pro-tumoral environment. Moreover, for the first time, we demonstrated up-regulation of the inhibitory molecule TIM3 in human monocytes after treatment with ATC cell-derived CM. More importantly, the TIM3 blockade partially reversed macrophage M2 polarization, demonstrating the modulation potential of the TIM3 pathway in ATC therapy (Figure 6G).

To date, only a few studies have explored the polarization and function of TAMs in thyroid cancer progression. In our study, the acquisition of a predominantly M2-like phenotype by the exposure of human monocytes to culture supernatants from thyroid tumor cells was comparable to that previously described by other research groups. In those studies, M2 macrophage markers were induced in primary monocytes after treatment with CM from tumor cells derived from different types of thyroid malignancies [55,56]. However, and unlike our results, Mazzoni et al. mention that some thyroid tumor cell-derived CM, including 8505c cell-derived CM, were not able to induce M2-like polarization of primary monocytes [56]. In our work, the tumor-promoting M2-like phenotype was robustly validated and confirmed through different methodologies, markers, and by the use of two BRAF-mutant cell lines derived from ATCs. Interestingly, our data are in line with a previous report showing the presence of M2-like macrophages infiltration in tumor xenografts derived from 8505c cells [57]. Therefore, the discrepancies between our results and those commented in the Mazzoni study can be explained by different reasons such as variations in experimental conditions and systems used, among others. In addition, Cho et al. reported that co-cultures of THP-1 cells or human monocytes with PTC cells increased the migration and invasion of PTC cells compared to PTC cells cultured alone [55]. Some studies have also reported that CM isolated either from macrophages exposed to CM from PTC cells or TAMs isolated from PTC tumors increased the migration [56] and invasion [58] of PTC cells, respectively. However, our present study is the first to our knowledge to investigate the impact of interactions between ATC cells and TAMs in thyroid cancer progression, with our results supporting these observations in PTC, and more importantly, our hypothesis that the phenotype and function of human monocytes can be adjusted by local cues secreted by tumor cells in ATC. Multiple signals released by the cancer cells, including cytokines, chemokines, and growth factors, would trigger the M2-like program. Further studies should be carried out in order to identify ATC cell-derived soluble factors involved in the activation of human monocytes toward an M2-like phenotype.

TAMs promote tumor progression by secreting growth factors and different cytokines, including IL-6. In agreement, our results showed that THP-1 cells after treatment with ATC cell-derived CM increased IL-6 levels, as detected by both RT-qPCR and ELISA assays (Figure 2). On the other hand, it has been described that STAT3 activation in TAMs drives polarization toward the M2 phenotype. Moreover, as activated STAT3 induces the super production of IL-6, we speculate that the increased activation of STAT3 may be involved in the high expression of IL-6. Therefore, we studied the effects of ATC cell-derived CM on the STAT3 pathway by Western blot and found that p-STAT3 expression was increased in human monocytes. Nevertheless, the question that remains unanswered is how STAT3 is phosphorylated in THP-1 cells. Related to this, several regulators have been reported to mediate the activation of STAT3, including IL-6, which in fact can be produced by tumor cells. In line with these observations, in a previous study, we detected the release of IL-6 by ATC cells [14]. Therefore, we hypothesized that the increased expression of IL-6 by ATC cells could drive the activation of STAT3 in human monocytes. Then, STAT3-activated M2 macrophages overexpressing IL-6 would promote thyroid cancer progression. In addition, IL-6 released by TAMs, in turn, would further activate the STAT3 pathway, forming a positive feedback loop between tumor and TME cells. Similarly, previous studies have also demonstrated the presence of this IL-6/STAT3 feedback loop in other types of cancer.

Interestingly, several combination therapies using signal transduction inhibitors have been tested for different types of tumors. A recent study demonstrated that the targeted inhibition of STAT3 in tumor-associated myeloid cells increases the efficacy of radiotherapy against head and neck squamous cell carcinoma by reducing M2 macrophages and triggering T cell-mediated anti-tumor immune responses [59]. Therefore, we speculate that the inhibition of IL-6/STAT3 signaling may have potential benefits in the treatment of ATC. However, further studies are needed to validate our hypothesis. Moreover, we cannot rule out the possibility that in addition to IL-6, other components of ATC cell secretome may also be inducing the activation of STAT3.

A detailed understanding of the complex interactions between tumor cells and TAMs is vital to identify potential therapeutic targets. Several macrophage-targeting strategies have been investigated, including TAMs depletion, inhibition of TAMs recruitment, suppression of pro-tumorigenic activity of TAMs, and reprogramming TAMs phenotype [18]. However, the crosstalk between ATC cells and TAMs is poorly understood. Here, we describe a paracrine loop between tumor cells and macrophages that promotes thyroid cancer. In order to identify the soluble mediators involved in the tumor-promoting effects of M2-like macrophages and therefore identify possible new opportunities for therapeutic intervention, we applied the cytokine array to CM from human monocytes after treatment with ATC cell-derived CM and found modifications in the release of several soluble proteins by THP-1 after treatment with ATC cell-derived CM. We next focused on the TIM3 protein, which was markedly up-regulated by the THP-1 cells after treatment with the CM derived from the two ATC cell lines. TIM3 is a negative regulator of immunity that is expressed in monocytes, macrophages, and other immune cells, where it plays an important role in maintaining immune homeostasis. Additionally, TIM3 has been reported to regulate the functions of monocytes and macrophages. However, its role in macrophages has not been reported in ATC. It has been demonstrated that TIM3 is highly expressed in M2 macrophages. Accordingly, by using flow cytometry, we detected a high expression of TIM3 in human monocytes promoted by ATC-cell derived CM. Next, we evaluated the effect of TIM3 on the polarization of macrophages in vitro. Blocking of TIM3 by the anti-TIM3 neutralizing monoclonal antibody resulted in a significant decrease in the expression of M2 macrophage markers as well as in IL-6 secretion, indicating that a high expression of TIM3 promotes macrophage polarization toward an M2-like phenotype in ATC. Recent advances in immunotherapy by using anti-checkpoint antibodies have transformed cancer therapy [60]. Despite the success of anti-PD-1/PD-L1 and CTLA-4 therapies, less of 20% of patients with ATC benefit from immunotherapy [61]. Currently, the therapeutic potential of anti-TIM3 antibodies is being investigated in clinical trials with promising results in cancer patients [50]. Therefore, TIM3 blockade might have great potential in ATC immunotherapy. Such possibility awaits future investigation.

However, it remains largely unclear what up-regulates TIM3 in human monocytes, as well as the molecular mechanism by which TIM3 regulates macrophage polarization. Yan et al. showed that an increase in the expression of TIM3 is induced by the transforming growth factor-β (TGF-β) produced by tumor cells [52], which facilitates the alternative activation of macrophages. We cannot exclude the possibility that TGF-β in the ATC TME was one of the molecules that can foster TIM3 up-regulation in macrophages. Whether TGF-β plays a role in TIM3 expression would be interesting to address in future studies. In fact, the present study did not identify the soluble factors secreted by ATC cells that can lead to an increased expression of TIM3. It was previously shown that TIM3 signaling increases PI3K–AKT phosphorylation in murine macrophages [62]. Furthermore, another study reported that TIM3 silencing decreases the phosphorylation of p65, which may indicate a role of TIM3 in the activation of NF-κB pathway [52]. However, the effects of TIM3 on the activation of AKT/NF-κB signaling were not evaluated here, with further studies being needed to explore the ATC cell-derived factors involved in TIM3 activation in human monocytes as well as to determine the mechanism by which TIM3 modulates macrophage polarization. Collectively, our present data identified possible new opportunities for therapeutic intervention.

## 5. Conclusions

In summary, our results provide valuable insights into the processes by which soluble factors produced by ATC cells induce the M2 polarization of human monocytes through TIM3. Moreover, and based on our data, TIM3 in TAMs should now be further evaluated as a possible potential novel target for treating this type of disease.

## Data Availability

The data presented in this study are available in the present manuscript and in the Supplementary Materials.

## Supplementary Materials

The following are available online at https://www.mdpi.com/article/10.3390/cancers13194821/s1, Figure S1: hIL-4 activates macrophages to a M2 phenotype. Figure S2: Effect of ATC cell-derived CM on inducible nitric oxide synthase (iNOS) expression and nitrite production in THP-1 cells. Figure S3: Human monocytes activated by ATC cell-derived CM increased STAT3 phosphorylation. Figure S4: Tumor-educated macrophages by soluble factors secreted by ATC cells exert tumor-promoting functions by increasing thyroid cancer cell migration. Figure S5: Human monocytes activated by ATC cell-derived CM increased the expression levels of MMP-9. Figure S6: M2 markers and TIM3 expression in human thyroid tissues. Figure S7: Uncropped Western Blots, Table S1.: Sequences of the primers used for PCR.
