# Supplementary material for: Secreted Factors by Anaplastic Thyroid Cancer Cells Induce Tumor-Promoting M2-like Macrophage Polarization through a TIM3-Dependent Mechanism
Source: Cancers (Basel). 2021 Sep 26;13(19):4821. doi: 10.3390/cancers13194821 (PMC8507981; doi:10.3390/cancers13194821)
Supplement: Supplementary file 1 [file cancers-13-04821-s001.zip › cancers-1387992-supplementary.pdf]

cancers

MDPI

Supplementary Material: Secreted Factors by Anaplastic Thyroid Cancer Cells Induce Tumor-Promoting M2-Like Macrophage Polarization through a TIM3-Dependent Mechanism

Cinthia Carolina Stempin, Romina Celeste Geysels, Sunmi Park, Luz Maria Palacios, Ximena Volpini, Claudia Cristina Motran, Eva Virginia Acosta Rodríguez, Juan Pablo Nicola, Sheue-yann Cheng, Claudia Gabriela Pellizas and Laura Fozzatti

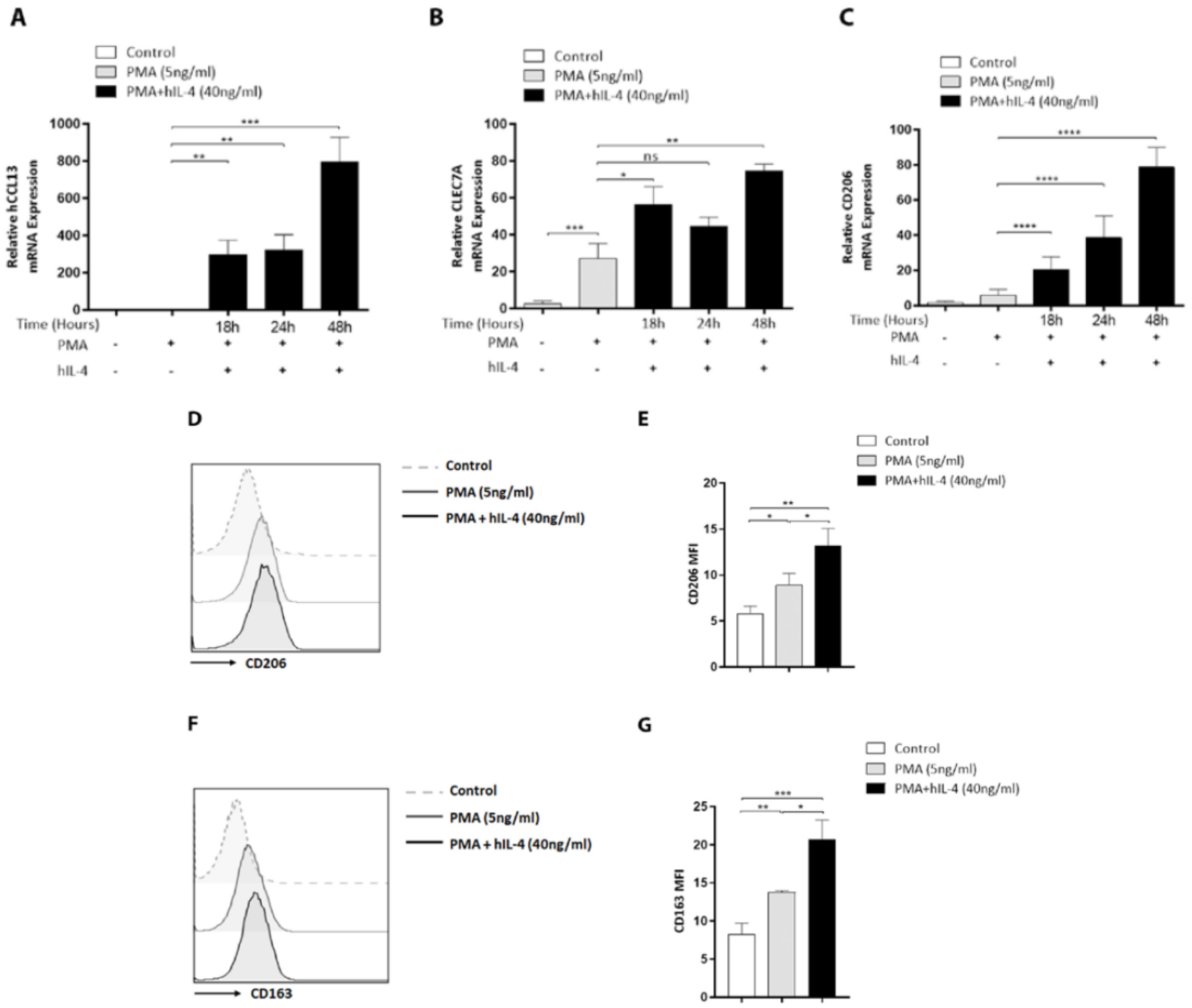

Figure S1. hIL-4 activates macrophages to a M2 phenotype. THP-1 cells treated with 5ng/ml PMA and 40ng/ml hIL-4 showed a significant increase of mRNA expression of hCCL13 (A), hCLEC7A (B) or CD206 (C) measured by RT-qPCR. (D-E) Flow cytometry analysis revealed a significant induction of CD206 in THP-1 cells treated with PMA and hIL-4 (D) compared to THP-1 control. Representative histograms (D) and quantification (E) are shown. (F-G) Flow cytometry analysis showed a significant induction of CD163 in THP-1 cells treated with PMA and hIL-4 (F) compared to THP-1 control. Representative histograms (F) and quantification (G) are shown. Data are expressed as mean ± SD. $*p<0.05$, $**p<0.005$, $***p<0.0005$, $****p<0.0001$. ns, not significant.

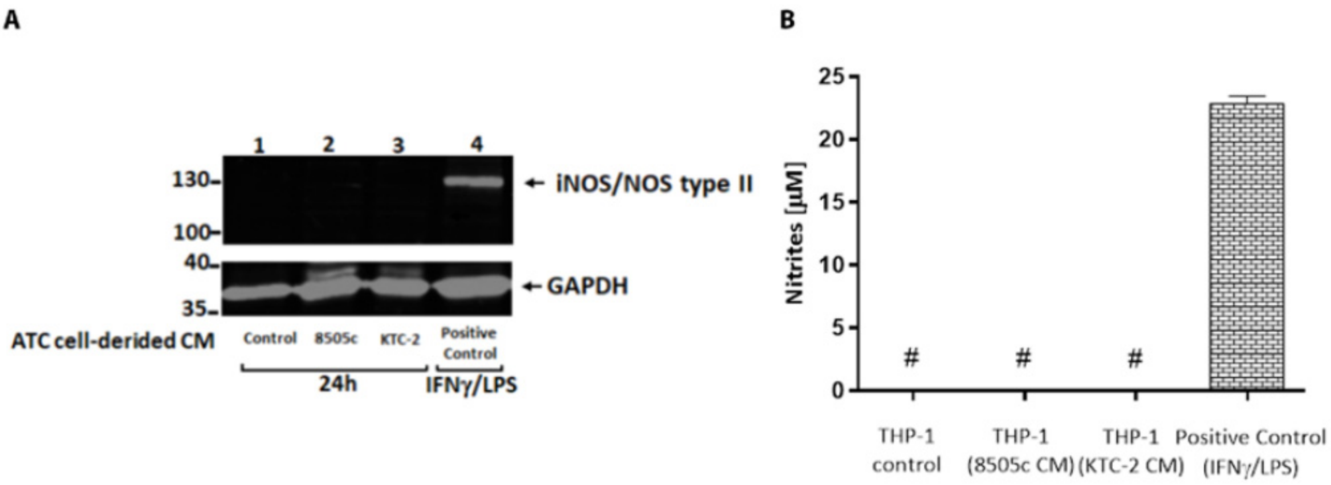

Figure S2. Effect of ATC cell-derived CM on inducible nitric oxide synthase (iNOS) expression and nitrite production, in THP-1 cells. (A, lanes 1 to 3) Immunoblot analysis of iNOS in THP-1 cells cultured in complete media containing 5% FBS (THP-1 control) or incubated with CM derived from ATC cells, 8505c [THP-1 (8505c CM)] and KTC-2 [THP-1 (KTC-2 CM)] for 24h. (A, lane 4) Western blot analysis of iNOS and GAPDH in macrophages treated with IFNγ + LPS for 24h, used as a positive control. (B) Comparison of nitrite production, measured by Griess reaction, in THP-1 cells cultured in complete media containing 5% FBS (THP-1 control) or incubated with CM derived from ATC cells, 8505c [THP-1 (8505c CM)] and KTC-2 [THP-1 (KTC-2 CM)] for 24h. Macrophages were stimulated with IFNγ + LPS for 24h and then subjected to Griess reagent assay. LPS, lipopolysaccharide. IFN (interferon) γ. #Not detectable.

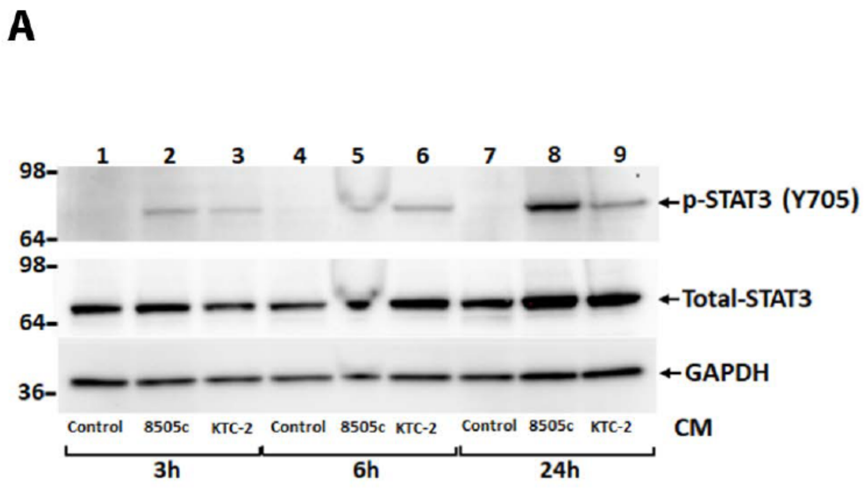

Figure S3. Human monocytes activated by ATC cell-derived CM increased STAT3 phosphorylation. Immunoblot analysis of STAT3 and phosphorylation in THP-1 cells cultured in complete media containing 5% FBS control (THP-1 control) and THP-1 cells treated with ATC cell-derived CM for the indicated time (A). The lower panel was the corresponding loading control using GAPDH.

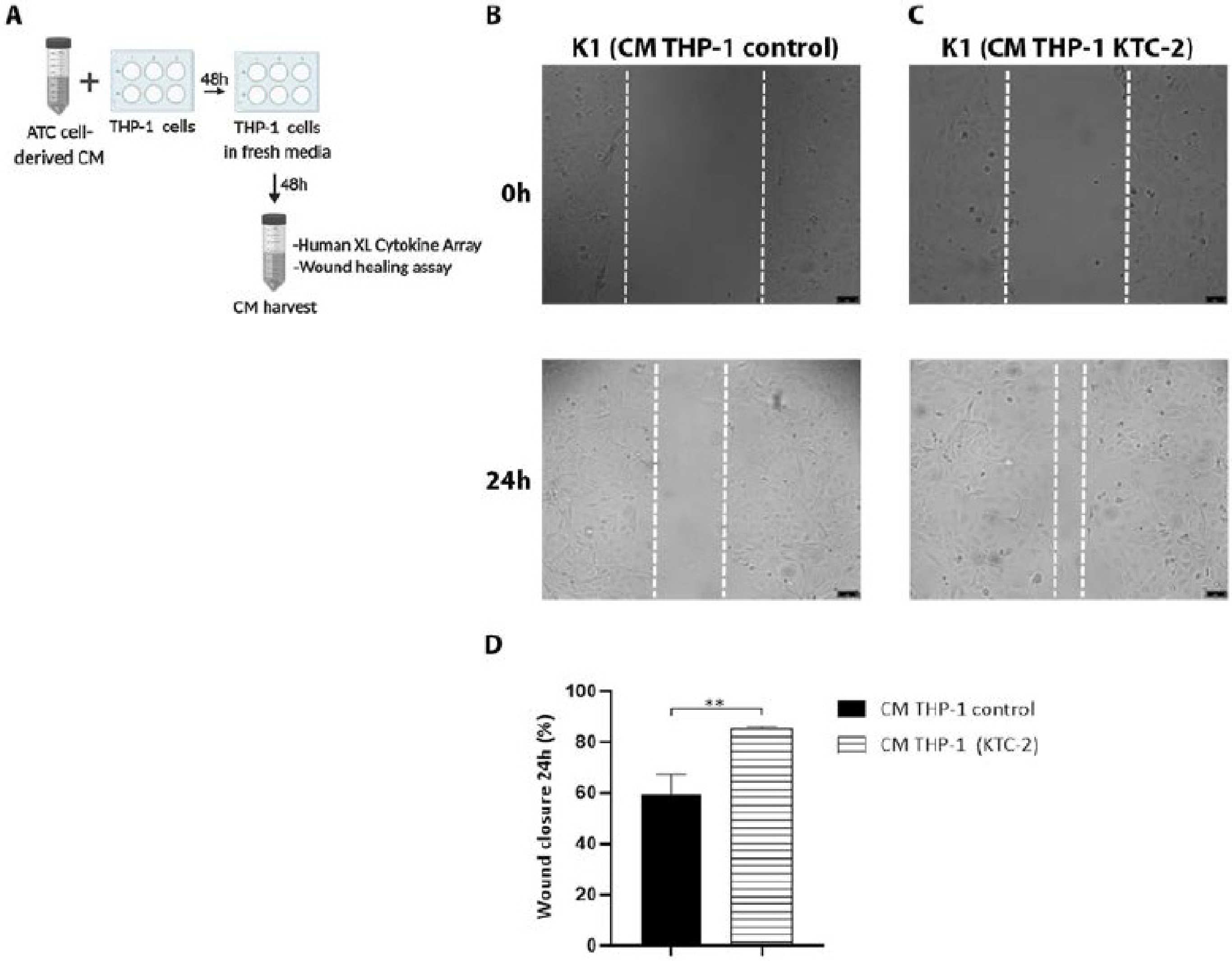

Figure S4. Tumor-educated macrophages by soluble factors secreted by ATC cells exert tumor-promoting functions by increasing thyroid cancer cell migration. (A) Complete media containing 5% FBS or 100% CM from KTC-2 cells were used for the treatment of THP-1 cells for 48h. The medium was discarded and then incubated with fresh medium. After 48h, 100% CM from THP-1 cells were collected and used to treat K1 cells. (B-C) Representative cell pictures of wound healing assay in K1 cells incubated with CM derived from THP-1 control (B) or CM derived from activated THP-1 (C). Quantitative analysis of wound-induced migration assay from B-C (D). Data are expressed as mean ± SD. $**p<0.005$. Created with BioRender.

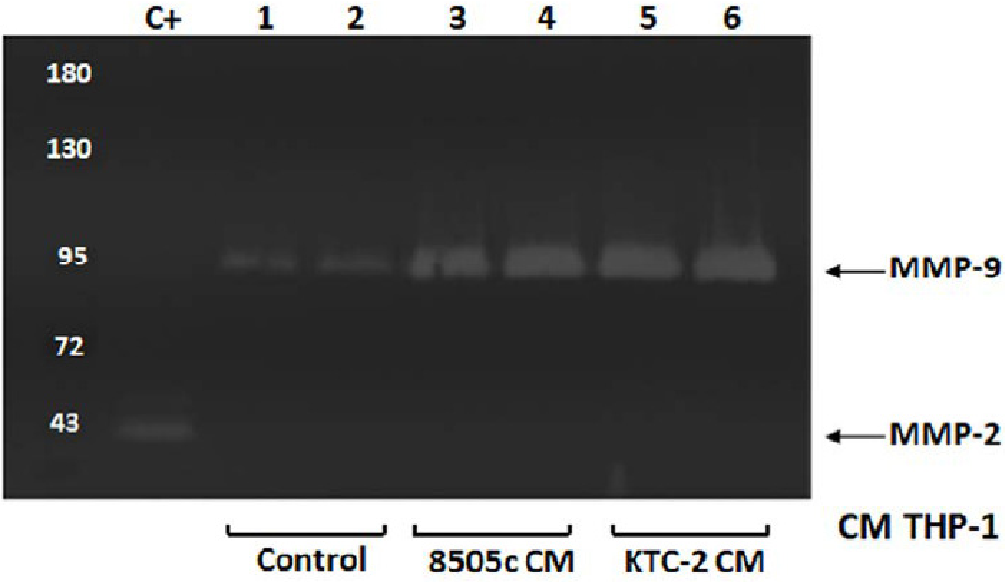

Figure S5. Human monocytes activated by ATC cell-derived CM increased the expression levels of MMP-9. The CM from THP-1 cells were prepared as described in figures 3F and 4A. Briefly, THP-1 cells were cultured with complete media containing 5% FBS or ATC cell-derived CM. After 48h, the medium was discarded and the cells were washed with PBS before being incubated with fresh medium containing 5% FBS. After 48h, the CM were harvested, centrifuged and used for gelatin zymography.

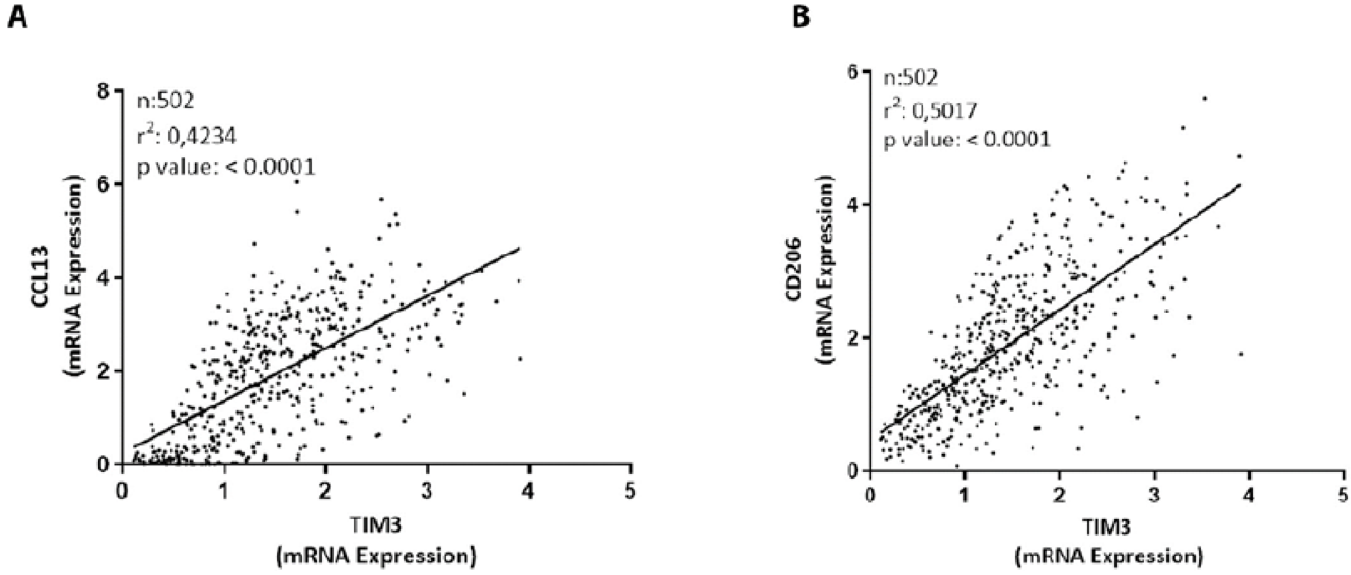

Figure S6. M2 markers and TIM3 expression in human thyroid tissues**.** Analysis of TCGA-THCA data. (A-B) Correlation between M2 markers (CCL13 and CD206) and TIM3.

Full-length gels uncropped Western Blots
Figure 1E Raw Data.

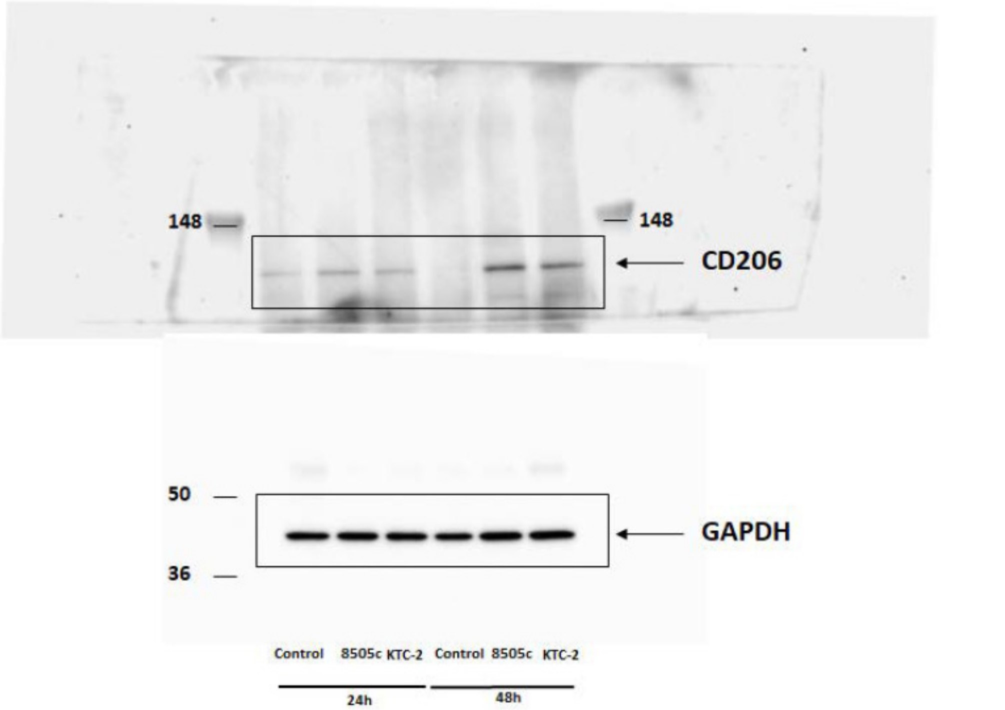

Figure 1J Raw Data.

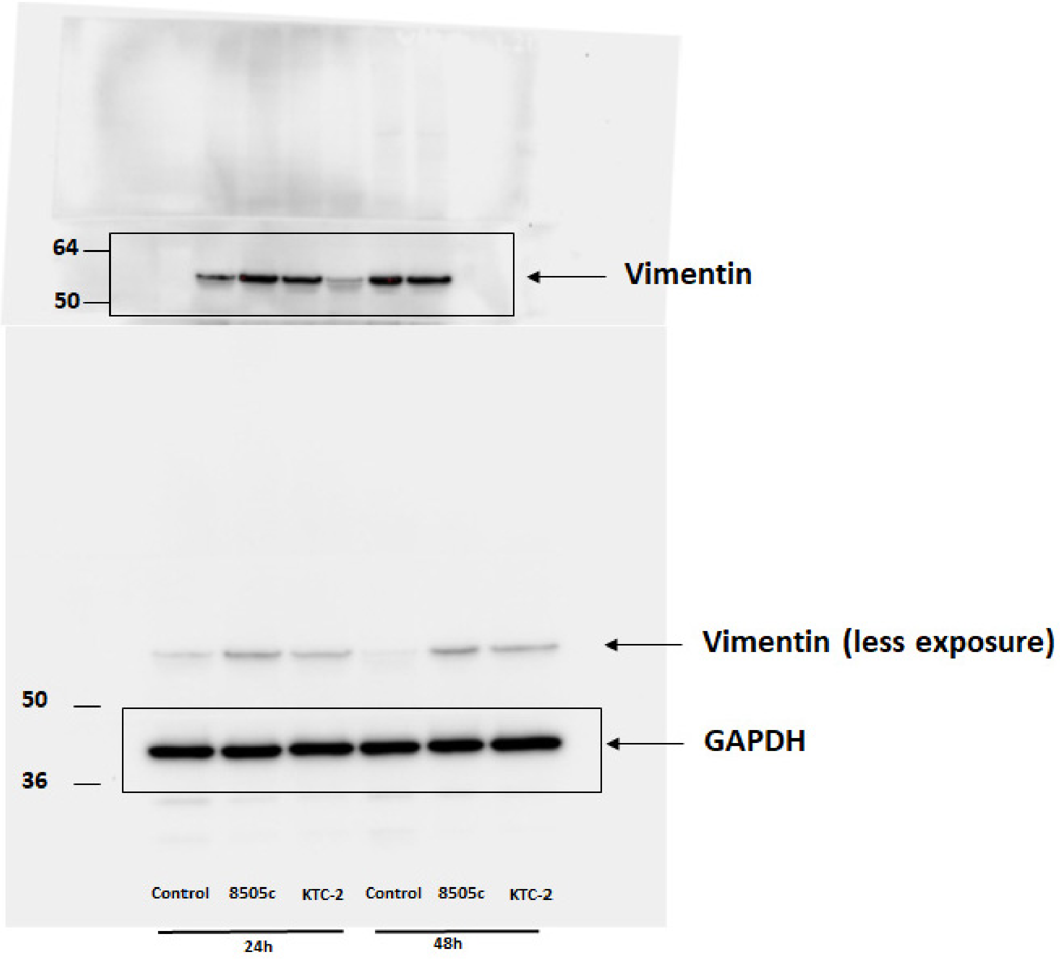

Figure 2D Raw Data.

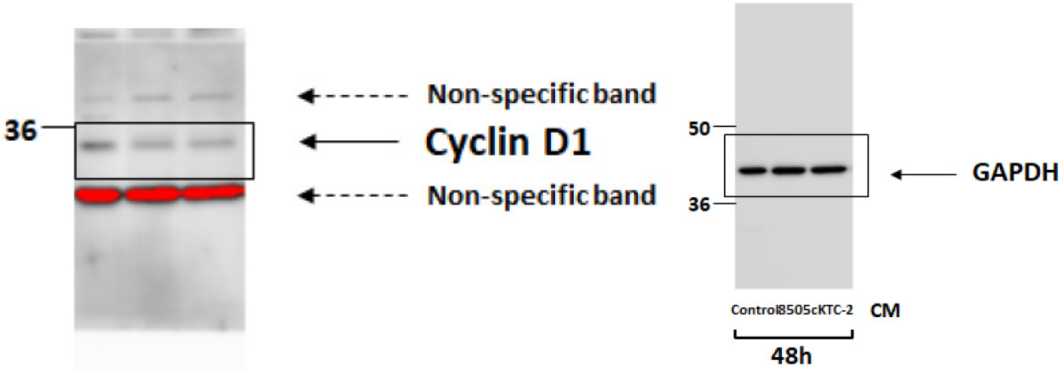

Figure 2F Raw Data.

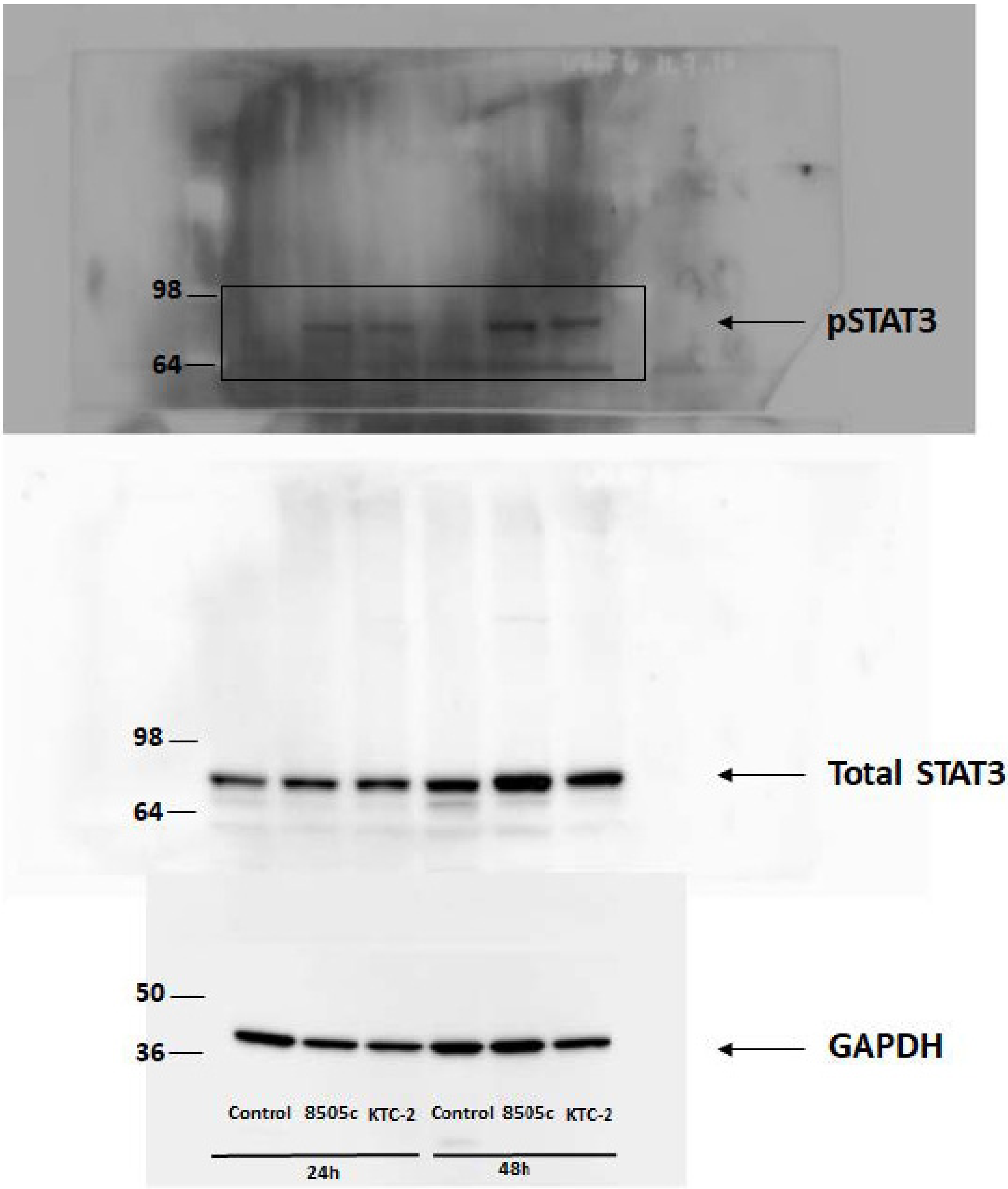

Figure S2 Raw Data.

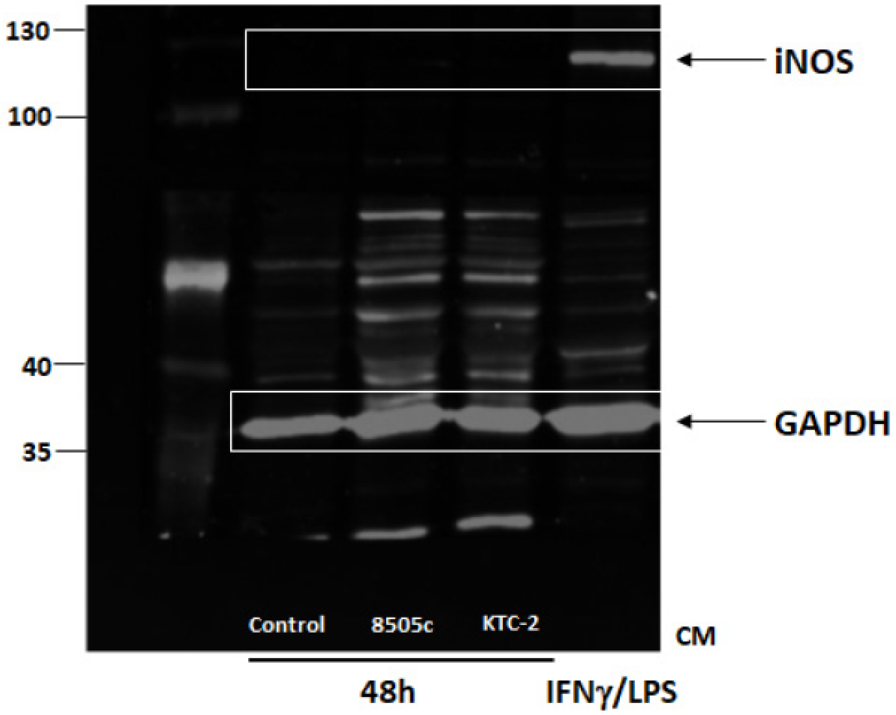

Figure S3 Raw Data.

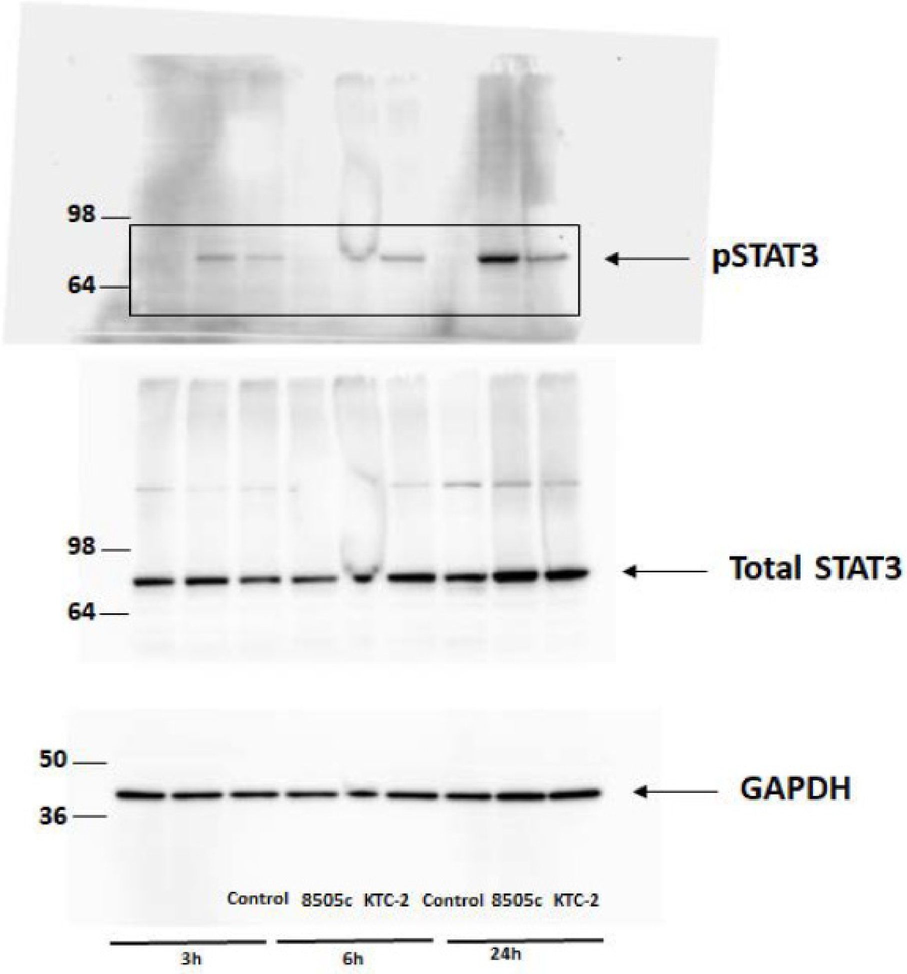

Figure 3J Raw Data.

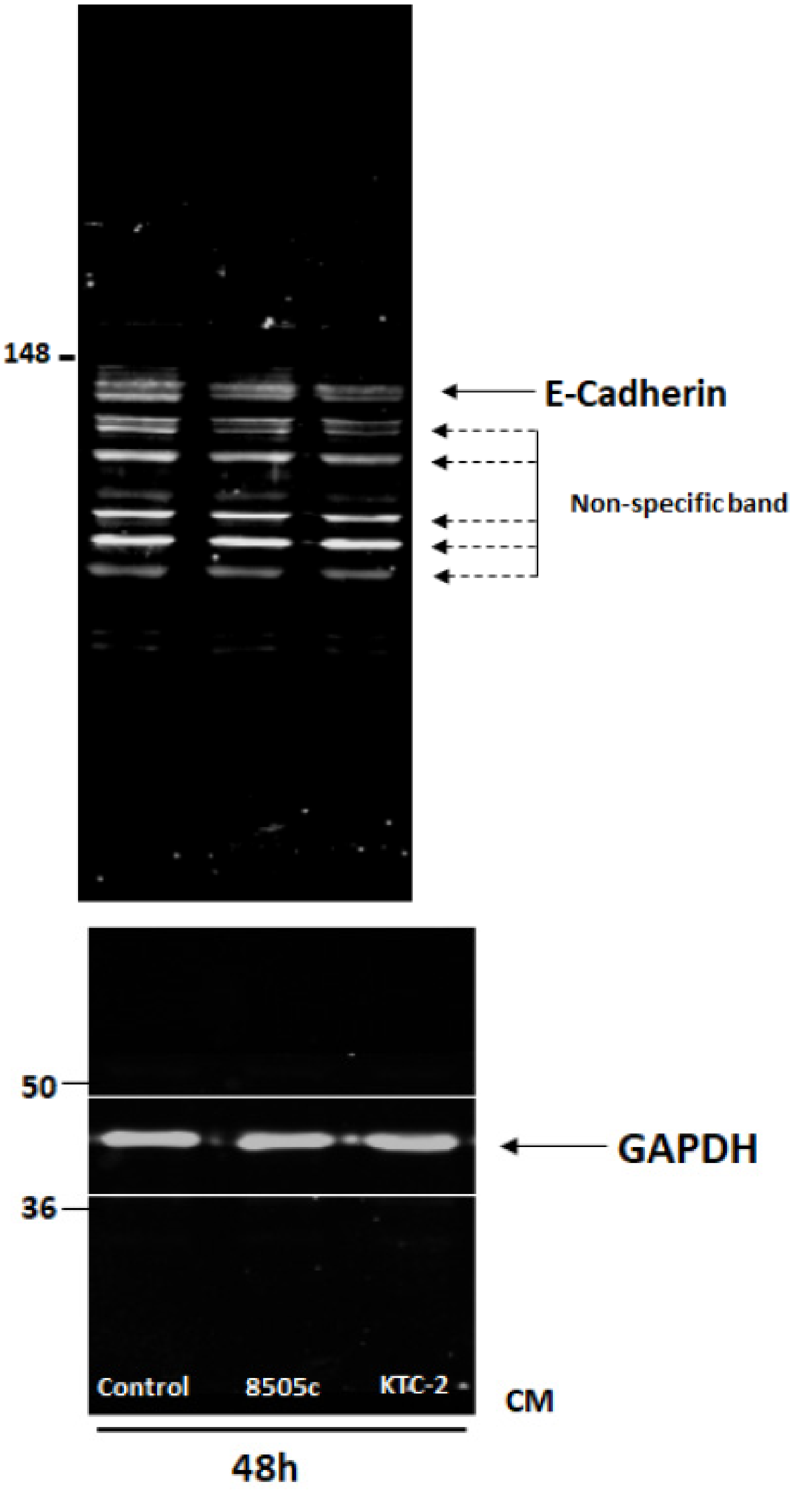

Figure S7. Uncropped Western Blots.

Table S1.: Sequences of the primers used for PCR.

	Forward Primer	Reverse Primer
hCD206	5’-GGCGGTGACCTCACAAGTAT-3’	5’-ACGAAGCCATTTGGTAAACG-3’
hCLEC7A	5’-AACCACAGCTACCCAAGAAAAC-3’	5’-GGGCACACTACACAGTTGGTC-3’
hCCL13	5’-AGCCAGATGCACTCAACGTC-3'	5’-TCTCCTTGCCCAGTTTGGTT-3'
hIL-6	5'-TCAATGAGGAGACTTGCCTG-3'	5'- CATCTGCACAGCTCTGGCT-3'
hGAPDH	5’-TTG TTG CCATCAATG ACCCCTT-3’	5’-CAGTGGACTCCACGACGTACTCAG-3’

h, human
